# Molecular Profiling of Multiple Human Cancers Defines an Inflammatory Cancer-Associated Molecular Pattern and Uncovers KPNA2 as a Uniform Poor Prognostic Cancer Marker

**DOI:** 10.1371/journal.pone.0057911

**Published:** 2013-03-25

**Authors:** Saleh M. Rachidi, Tingting Qin, Shaoli Sun, W. Jim Zheng, Zihai Li

**Affiliations:** 1 Department of Microbiology and Immunology, South Carolina Clinical and Translational Research Institute (SCTR), Medical University of South Carolina, Charleston, South Carolina, United States of America; 2 Hollings Cancer Center, South Carolina Clinical and Translational Research Institute (SCTR), Medical University of South Carolina, Charleston, South Carolina, United States of America; 3 Division of Bioinformatics, Department of Biochemistry and Molecular Biology, South Carolina Clinical and Translational Research Institute (SCTR), Medical University of South Carolina, Charleston, South Carolina, United States of America; 4 Department of Pathology and Laboratory Medicine, South Carolina Clinical and Translational Research Institute (SCTR), Medical University of South Carolina, Charleston, South Carolina, United States of America; 5 Computational Biology Core Facility, South Carolina Clinical and Translational Research Institute (SCTR), Medical University of South Carolina, Charleston, South Carolina, United States of America; Baylor College of Medicine, United States of America

## Abstract

**Background:**

Immune evasion is one of the recognized hallmarks of cancer. Inflammatory responses to cancer can also contribute directly to oncogenesis. Since the immune system is hardwired to protect the host, there is a possibility that cancers, regardless of their histological origins, endow themselves with a common and shared inflammatory cancer-associated molecular pattern (iCAMP) to promote oncoinflammation. However, the definition of iCAMP has not been conceptually and experimentally investigated.

**Methods and Findings:**

Genome-wide cDNA expression data was analyzed for 221 normal and 324 cancer specimens from 7 cancer types: breast, prostate, lung, colon, gastric, oral and pancreatic. A total of 96 inflammatory genes with consistent dysregulation were identified, including 44 up-regulated and 52 down-regulated genes. Protein expression was confirmed by immunohistochemistry for some of these genes. The iCAMP contains proteins whose roles in cancer have been implicated and others which are yet to be appreciated. The clinical significance of many iCAMP genes was confirmed in multiple independent cohorts of colon and ovarian cancer patients. In both cases, better prognosis correlated strongly with high *CXCL13* and low level of *GREM1, LOX, TNFAIP6, CD36*, and *EDNRA*. An “Inflammatory Gene Integrated Score” was further developed from the combination of 18 iCAMP genes in ovarian cancer, which predicted overall survival. Noticeably, as a selective nuclear import protein whose immuno-regulatory function just begins to emerge, karyopherin alpha 2 (KPNA2) is uniformly up-regulated across cancer types. For the first time, the cancer-specific up-regulation of KPNA2 and its clinical significance were verified by tissue microarray analysis in colon and head-neck cancers.

**Conclusion:**

This work defines an inflammatory signature shared by seven epithelial cancer types and KPNA2 as a consistently up-regulated protein in cancer. Identification of iCAMP may not only serve as a novel biomarker for prognostication and individualized treatment of cancer, but also have significant biological implications.

## Introduction

The relationship between cancer and inflammation was observed as early as the 19^th^ century, when Ronald Virchow described tumor-infiltrating leukocytes. However, it was not until the past two decades that the inflammatory microenvironment has been recognized as a key component in carcinogenesis, being involved in cancer initiation, promotion and metastasis [1]. Chronic infections are established etiological factors for many human cancers [2]. Similarly, chronic inflammation such as inflammatory bowel disease and chronic hepatitis increases the risk for colorectal and hepatocellular carcinomas, respectively. In most cases, immune-surveillance is thought to eliminate tumorigenic foci [3]. However, cancer-initiating cells reprogram immune cells to create a tumor-friendly microenvironment, thereby evading antitumor immunity. In addition, cancer can educate both the innate and adaptive arms of the immune system through myeloid-derived suppressor cells [4], regulatory T cells [5] and other mediators, to promote growth and invasion.

The role of inflammation in carcinogenesis begins with tumor initiation, through multiple mechanisms such as genotoxic stress via reactive oxygen species, induction of activation-induced cytidine deaminase (AID) [6], TNF-α-induced entry of β-catenin into the nucleus [7] and others. Beyond initiation, cytokines activate pro-tumorigenic transcription factors such as STAT3 and NF-κB in existing cancer cells [8]. Inflammatory cells also dampen antitumor immunity through molecules such as indoleamine-2,3-dioxygenase and arginase 1, which interfere with the function of T lymphocytes [4]. Epithelial-mesenchymal transition is also favored by cytokines such as TGF-β, promoting distal metastasis [9].

“Onco-inflammation” therefore contributes to the different cancer hallmarks, including cell proliferation, angiogenesis and escape from apoptosis. However, despite this elaborate cross-talk between cancer cells and the inflammatory microenvironment, the approach to uncover critical players in this interaction has so far been sporadic and non-comprehensive. In this study, we hypothesize that a common inflammatory pattern exists among different cancer types, constituting a signature profile termed as iCAMP. We performed a comprehensive analysis of gene expression across 7 common epithelial cancer types. A robust oncoinflammatory profile was identified, demonstrating independent and strong predictive values in clinical outcomes of multiple cancers. This approach has also led to the discovery and validation of KPNA2 as the single most consistently up-regulated protein in cancer.

## Methods

### Ethics Statement

Access to patient samples and anonymous analysis of data was approved by the Institutional Review Board for Human Research at the Medical University of South Carolina.

### Datasets

This analysis included gene expression profiles from 7 types of cancer. One dataset was included for each cancer type, resulting in a total of 7 datasets. The cancers included in this study are: breast, colon, lung, oral, prostate, pancreatic and gastric cancers [10], [11], [12], [13], [14], [15], [16]. Datasets were obtained from Gene Expression Omnibus (GEO) Datasets, an NCBI public database. Each of the datasets included microarray mRNA expression data from cancer and normal tissue (Table S1). All of the datasets used [HG-U133_Plus_2] Affymetrix Human Genome U133 Plus 2.0 Array platform for quantification of gene expression levels.

### CDEP analysis on seven GEO datasets

The seven epithelial cancer types were investigated to identify genes showing consistent differential expression using the recently developed Consistent Differential Expression Pattern (CDEP) method [17]. The raw microarray data comparing gene expression between normal and cancer cells was downloaded from NCBI's GEO database (http://www.ncbi.nlm.nih.gov/gds/) (Table S1). After excluding 10 adenoma samples in the colon dataset, 545 samples were studied, of which 324 were from cancer tissue and 221 were normal. The expression values of the replicate samples in the pancreatic cancer dataset were averaged. The raw datasets were pre-processed individually. For each dataset, gene expression values were adjusted and normalized by the GCRMA approach implemented in R [18]. The false discovery rate (FDR) of each gene on each normalized dataset was calculated using permutation method implemented by the “RankProd” package in R [19]. Each gene in each dataset was then associated with a raw FDR, 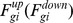 for being up/down-regulated [17]. For genes not present in the platform of a dataset, the median FDR value of that dataset was assigned [17].

CDEP meta-analysis was then applied to the raw FDRs of the seven datasets. For each dataset, the false positive rate is defined as the probability of a non-up-regulated gene being falsely called as over-expressed (or a non-down-regulated gene being falsely called as repressed). The number of genes being up-regulated, down-regulated, and non-differentially expressed for each dataset was estimated by a Beta mixture model implemented in WinBUGS [20]. Based on this rate and using independent Bernoulli distributions, we calculated the likelihood of a gene to be falsely identified as over/under-expressed among the seven datasets for each FDR threshold *l.* The procedure was evaluated by estimating the false discovery rate (FDR_g_) observing the above expected log likelihood, i.e. the proportion of false positives among the genes identified to be consistently differentially expressed. The “null log likelihood” was computed by permuting the 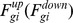
values relative to the genes within each dataset, and then performing the same above procedures to calculate the expected value of the “null log likelihood” in each permutation *b* for every gene, using 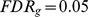 as a cutoff.

### Database for Annotation, Visualization and Integrated Discovery (DAVID)

After identifying the differentially expressed genes across the 7 cancer types, the set of up-regulated and down-regulated genes was entered into DAVID database (http://david.abcc.ncifcrf.gov/), and those with gene annotations related to inflammation and/or immune response were selected for further analysis.

### Human Protein Atlas (HPAT)

Six of the seven cancer types and their corresponding normal tissue were investigated for the protein expression level of all of the immune-related genes by HPAT (www.proteinatlas.org).

### Oncomine Cancer Database

Oncomine database (www.oncomine.org) was used to identify the clinical significance of the immune-related genes and their ability to predict patient survival and disease recurrence. X-tile software [21] was used to determine the optimal cut-off points for separating low risk from high risk patients.

### Inflammatory Gene Integrated Score (IGIS)

IGIS for each ovarian cancer patient is the summation of the risk value of 18 iCAMP genes with independent prognostic significance demonstrated by the training dataset [22]. These genes are *CCL28, CXL12, EDNRRB, GFRA1, GREM1, IL8, JAM2, LOX, MAL, MIF, MPZL2, PIGR, PTGER4, RSAD2, SERPINA5, TFF3, TNFAIP6 and TNFSF4*. The predictive value of IGIS was tested using an independent TCGA ovarian cancer dataset, based on cut-off values pre-determined by the training dataset [22]. For a given patient, the value of a gene conferring poor prognosis is its relative risk (RR), whereas the value of a gene whose expression in that patient predicts better prognosis was set as zero. As an example, if a patient A falls in the high risk group for genes 1 and 2 but in the low risk group for gene 3, this patient's IGIS will be the summation of Relative Risk for gene 1 and Relative Risk for gene 2, since the value of gene 3 is zero.

### Ingenuity Pathway Analysis (IPA)

Using the Ingenuity Pathway Analysis tool (www.ingenuity.com), inflammatory genes were mapped into multiple networks, each showing over-expressed and under-expressed ones.

### Tissue Microarrays

Tissue microarrays (TMAs) for colon cancer and head-neck cancer specimens contained formalin-fixed, paraffin embedded tissues. Colon cancer TMAs were developed from patient specimens obtained at the Medical University of South Carolina (MUSC), Charleston, SC, USA. Our study was approved by the Institutional Review Board. The colon cancer cohort consisted of tumors from 55 patients and adjacent normal tissue from 50 of them, along with 15 lymph node metastatic samples. Each of the normal and cancer specimens was at least in duplicate. Clinical and demographic information including age, sex, histologic type, grade, tumor (T) and lymph node (N) stages, overall and recurrence-free survival were obtained from the Cancer Registry of the Hollings Cancer Center at the Medical University of South Carolina. The head-neck cancer TMA was obtained from US Biomax, which contained 60 head-neck squamous cell carcinomas and normal specimens from 9 independent patients.

### Immunohistochemistry (IHC)

5-μm sections were cut from the TMA blocks. KPNA2 staining was performed using a rabbit polyclonal antibody specific to human KPNA2 (Abcam, Cat # 84440) at 1∶500 dilution. Slides were baked for 1 hour at 60°C and de-paraffinized. After antigen retrieval using citrate buffer (pH = 6.0), endogenous peroxidase was quenched using 3% H_2_O_2_ in dH_2_O for 5 minutes and non-specific binding was blocked by 2% normal goat serum for 3 hours at room temperature. Samples were incubated with anti-KPNA2 antibody at 4°C for 16 hours, followed by secondary antibody (Vectastain ABC Kit, PK-4001) and development using DAB substrate (Vector Labs SK-4100). Staining showed absolute specificity to the nucleus without discernible off-target signal. KPNA2 quantification was performed by a surgical pathologist (S.S.) who was blinded to the patient's clinical parameters. Quantification included nuclear staining intensity (1: faint; 2: moderate; 3: strong but less intense than 4; and 4: intense) and percentage of positive nuclei over all tumor cells in one TMA core.

## Results

### Data-mining workflow and identification of consistently differentially expressed genes in seven human cancer types

**Figure 1:**
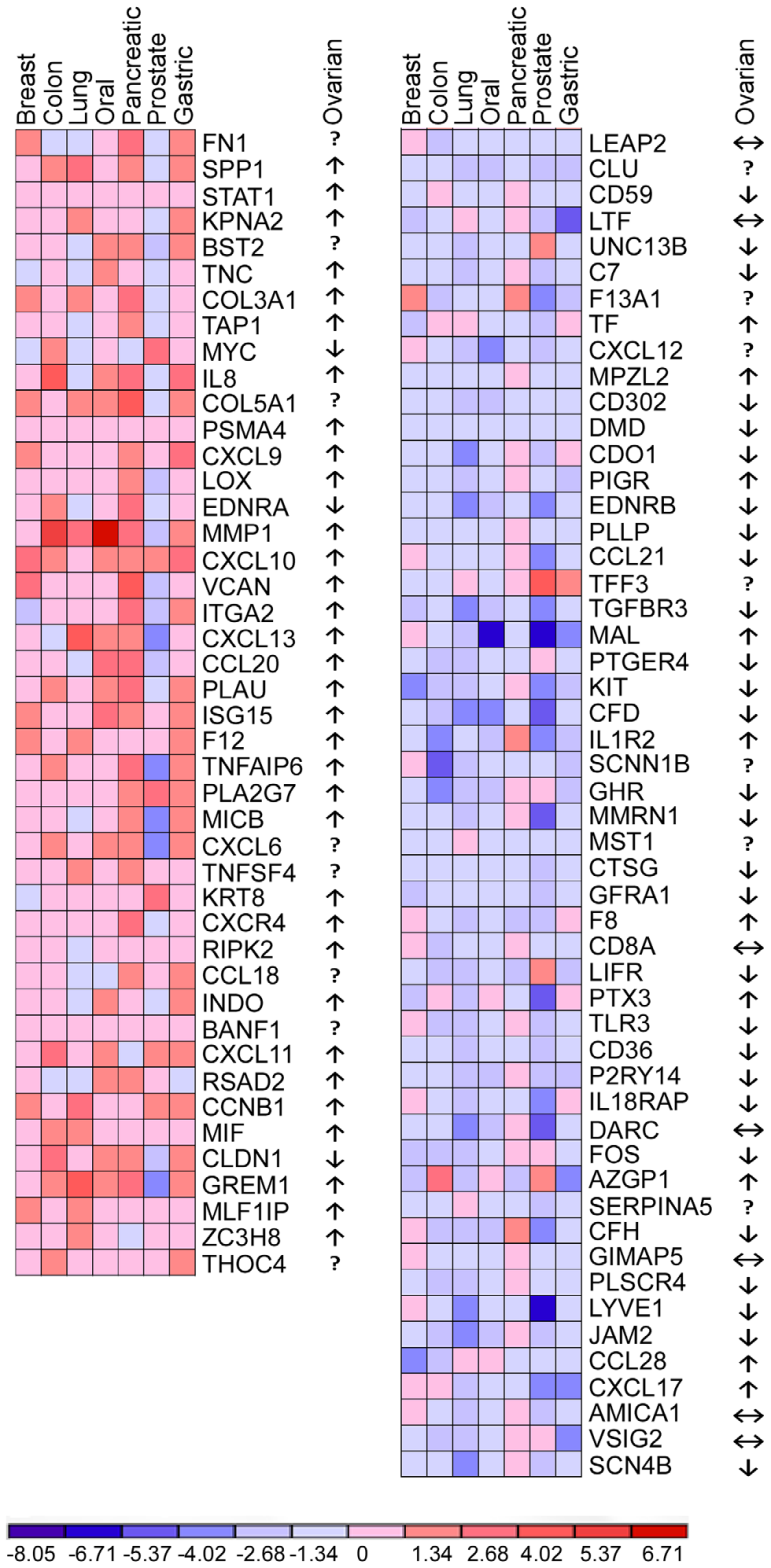
Heat map of dysregulated inflammatory genes. Heat map showing the 44 up-regulated and 52 down-regulated genes across the seven cancer types, in addition to the direction of dysregulation in ovarian cancer.↑, up-regulated.↓, down-regulated. ↔, unchanged.

Raw data from 7 datasets (Table S1) was first obtained from Gene Expression Omnibus (GEO). Each dataset corresponds to one cancer type: breast, colon, lung, oral, pancreatic, prostate and gastric cancers. These datasets include expression microarray profiles from cancer tissues and corresponding normal tissues. After determining dysregulated genes in each cancer type, the Consistent Differential Expression Pattern (CDEP) methodology was implemented to identify differentially expressed genes across the seven datasets. 911 genes were up-regulated and 618 genes were down-regulated. Using DAVID, these genes were then functionally classified and the immune-related genes were identified (Figure S2, Table S2). To further improve the signal-to-noise ratio, genes which showed a fold change (FC) <2 in five or more cancer types were excluded. This resulted in a robust inflammatory profile, termed iCAMP, of 44 up-regulated and 52 down-regulated genes at the mRNA level (Figure 1). The protein expression of iCAMP was verified by IHC in 6 cancer types: breast, colon, lung, pancreatic, prostate and gastric by mining the Human Protein Atlas (Table S3, Figure S5). The clinical significance of iCAMP was determined by using Oncomine database. Finally, the Ingenuity Pathway Analysis tool was used to pinpoint the function of iCAMP in cancer-associated inflammation.

### Identification of immune-related genes

Among the up-regulated genes in iCAMP, those involved in the positive regulation of lymphocyte apoptosis were significantly enriched by 11.2 folds. Small chemokines of the CXC family, such as *CXCL9*, *CXCL10* and *CXCL11* were enriched by 9.5 folds (Figure S2A). Additionally, tumors were also enriched with genes in other immunological categories such as host-virus interaction and response to wounding (Figure S2A). Down-regulated genes were highly enriched for complement components in the alternative pathway (9.9 folds), T-cell-associated genes such as CD8α, granzyme A and mal, T cell differentiation proteins (7.3 folds) and genes involved in regulating NK cell function such as lectin-like receptor subfamily K, member 1 (*KLRK1*) (Figure S2B).

The functions of many iCAMP genes are yet to be understood. For example, KPNA2 was shown to be upregulated across a broad-spectrum of cancer types (Figure 1) [23], [24], [25], [26], [27]. It is unclear, however, if the possible tumor-promoting activity of KPNA2 is due to its function as a nuclear transporter for selective immunoregulatory proteins such as STAT1 [28] and interferon-γ-induced transcription factor IRF-1 [29].

### Expression of iCAMP at the protein level

**Figure 2:**
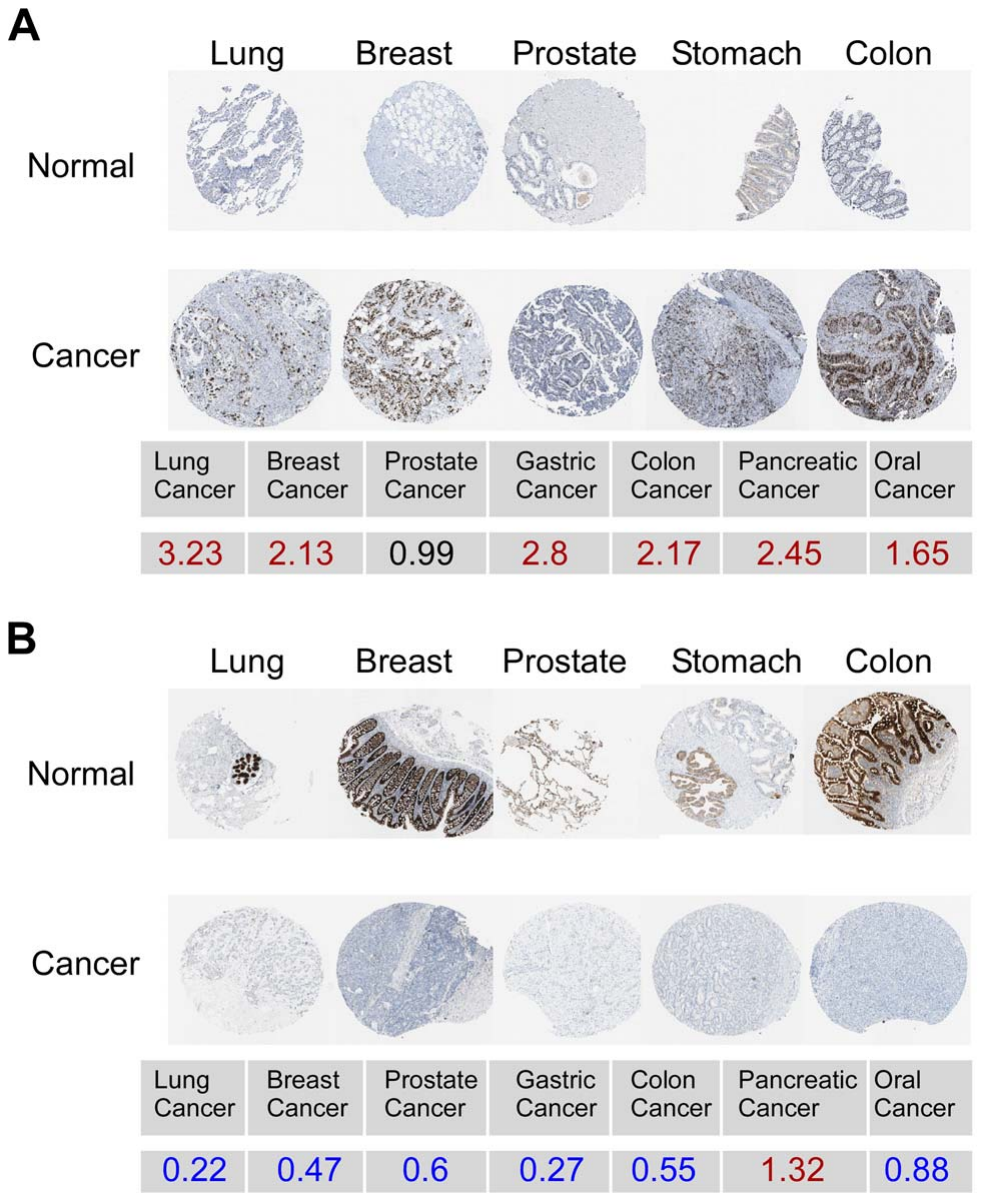
Expression of KPNA2 and PIGR in multiple cancer types. An example of an up-regulated gene (KPNA2) (A) and a down-regulated gene (PIGR) (B) in 5 cancer types on the protein level. Numbers correspond to average fold changes of the mRNA levels across all 7 cancer types in the current study.

To examine the protein expression pattern of the iCAMP genes, we took advantage of the publically available protein expression database, HPAT. Genes which did not show differential expression by IHC were not excluded due to the limited sensitivity of this method. Using IHC as a filtering step would expand the false negative observations, limiting the power of this study. However, it was informative to define which genes showed protein changes in what cancer types as this has direct implications on the choice of genes for further functional analyses. For example, KPNA2 was found to be increased in all cancer types except pancreatic cancer (Figure 2). On the other hand, polymeric immunoglobulin receptor (PIGR), a protein involved in the trans-epithelial transport of immunoglobulins, is consistently down-regulated in cancer cells (Figure 2). Altogether, protein expression data is available for 34 up-regulated and 38 down-regulated genes (total  = 72) of the 96 genes (Table S3, Figure S5). Of the 34 up-regulated genes at the mRNA level, 13 were also up-regulated by IHC in at least 3 of 6 cancer types studied. As for the transcriptionally down-regulated genes (38 were examined for protein expression), 20 showed down-regulation by IHC in ≥3 of the same 6 cancer types.

### Clinical significance of iCAMP genes

We next chose to use ovarian cancer to study the significance of iCAMP genes based on two considerations. First, most of ovarian malignancies are of epithelial origin which is histologically similar to the seven cancer types from which the gene profile was defined. Second, ovarian cancer was not used to generate the iCAMP genes. The predictive value of these genes in ovarian cancer would validate the utility of our gene discovery approach. As a control, the clinical predictive values of these genes were also examined in colon cancer. To determine the direction of dysregulation of each gene in ovarian cancer, we mined 5 published datasets [30], [31], [32], [33], [34] and one additional dataset from The Cancer Genome Atlas (TCGA) (Table S4), which collectively contain 35 normal and 878 cancer samples. Based on the number of datasets showing dysregulation in each direction, each gene was labeled as elevated, unchanged or repressed in ovarian cancer (Figure 1). A gene is determined to be elevated if 1) at least 1 dataset shows up-regulation (p<0.05) and none of the other datasets shows down-regulation, or 2) at least 3 datasets show up-regulation and not more than one dataset shows down-regulation (p<0.05). The inverse applies to repressed genes. Based on that, 33 of the 44 up-regulated genes were also elevated in ovarian cancer, 3 were repressed and 8 could not be determined. Among the 52 down-regulated genes, 28 were also repressed in ovarian cancer, 7 were unchanged, 10 were elevated and 7 were undetermined (Figure 1). Thus, at least 61 out of 96 iCAMP genes were concordantly dysregulated in ovarian cancer.

**Figure 3:**
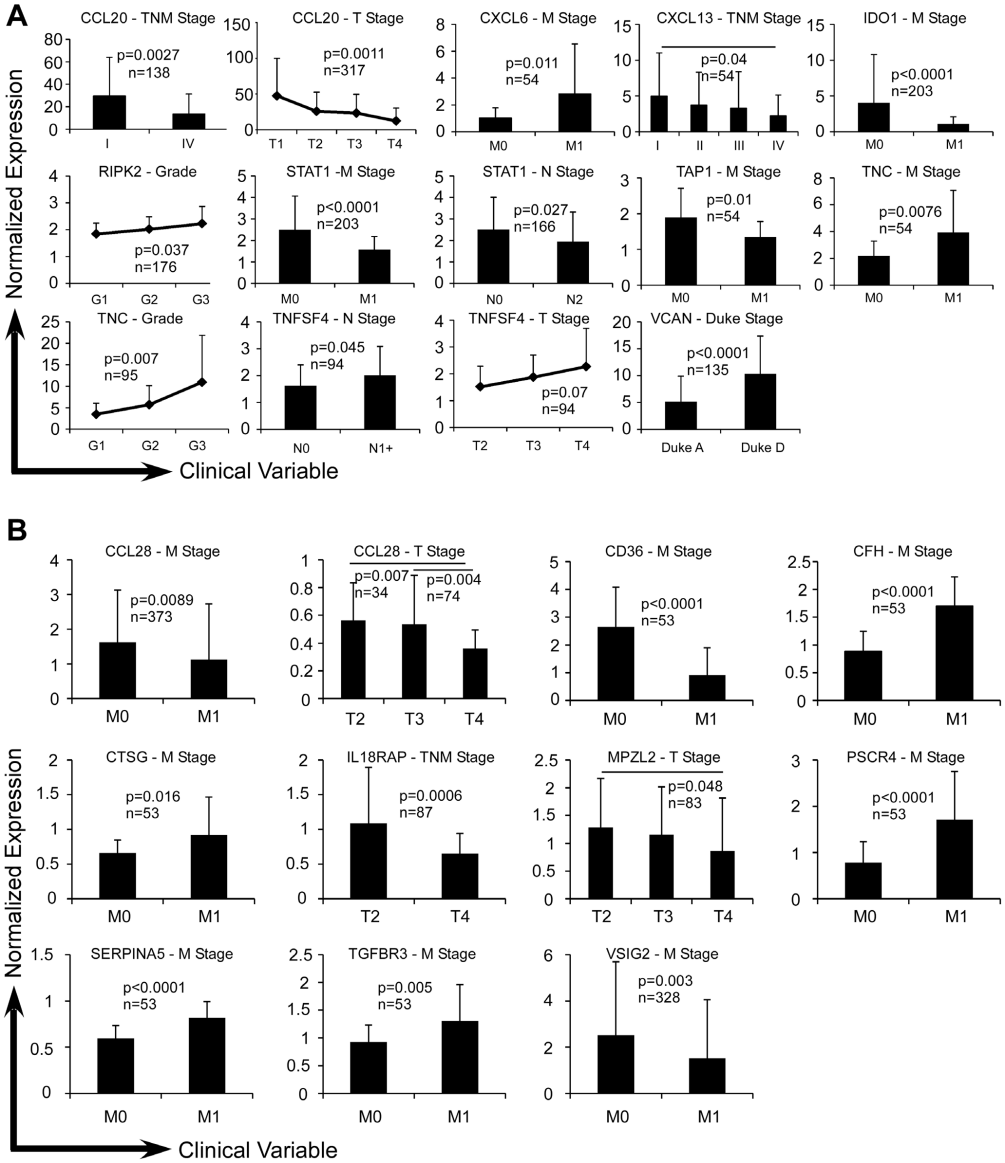
Up-regulated (A) and down-regulated (B) genes with clinical correlations in colon cancer. Four independent studies were analyzed for the expression of indicated genes and their clinical significance. The vertical axis represents the normalized expression intensity of each gene relative to the median intensity of the entire gene probes. Error bars represent standard deviation. Genes are listed in alphabetical order.

**Figure 4:**
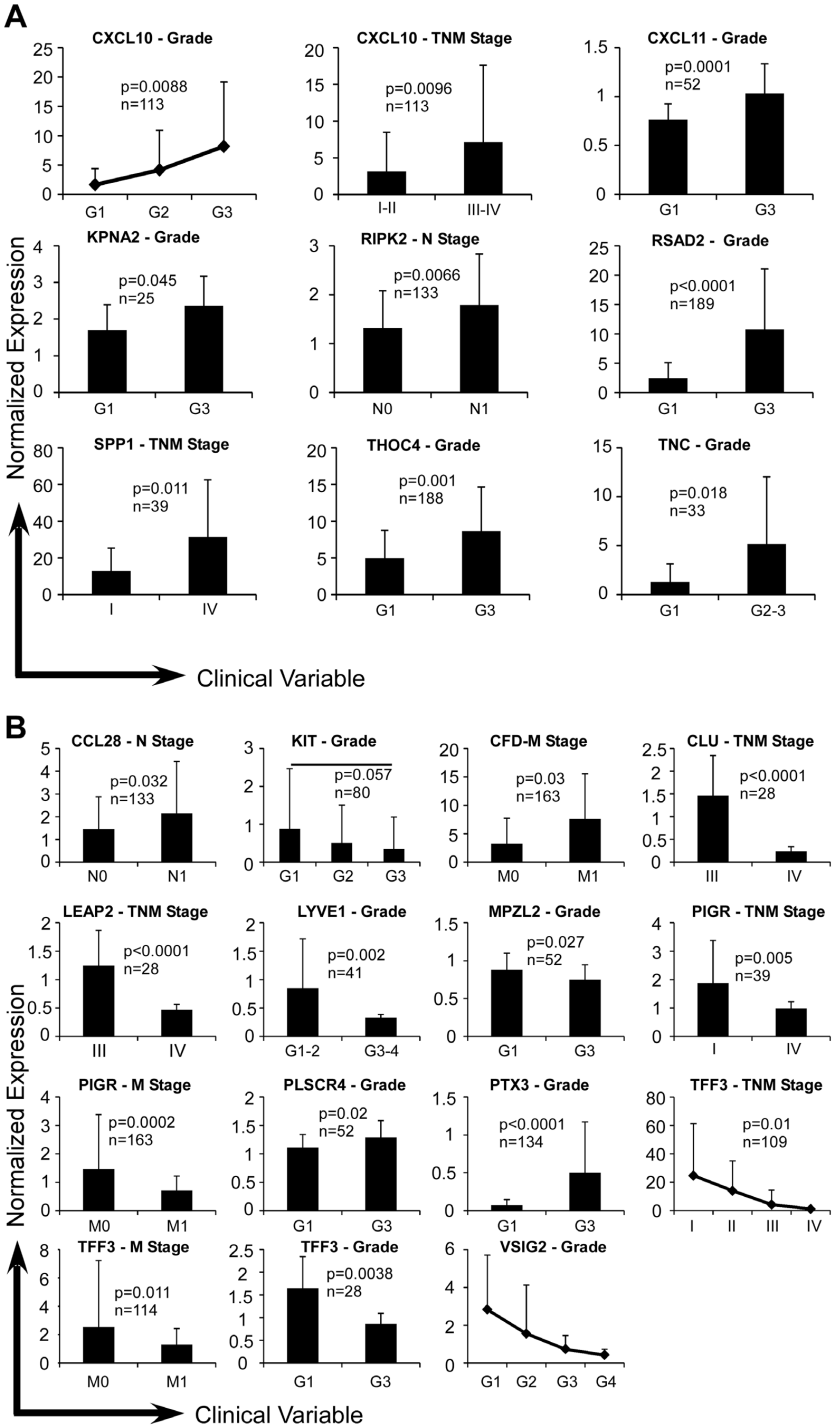
Up-regulated (A) and down-regulated (B) genes with clinical correlations in ovarian cancer. Same as in Figure 3 except that five independent studies of ovarian cancer were analyzed for the expression of indicated genes and their clinical value.

Colon [35], [36], [37] and ovarian cancer datasets [22], [30], [38], [39], [40] from Oncomine, as well as TCGA colon cancer dataset, were then analyzed for clinical significance of expression level of individual iCAMP gene. A number of genes such as *CCL20*, *CD36* and *IL18RAP* individually showed significant correlations with clinical variables in colon cancer such as tumor stage (T1 to T4), lymph node status (N0 to N2), metastasis (M0 or M1), pathological grade (G1 to G4) and Duke stage (A to D) (Figures 3A and 3B). In ovarian cancer, all of the up-regulated genes were increased with more advanced stage (*CXCL10*, *RIPK2* and *SPP1*) or higher pathological grade (*CXCL11*, *KPNA2*, *RSAD2*, *THOC4* and *TNC*) (Figure 4A). As for the down-regulated iCAMP genes, more advanced stages conferred higher expression of some genes (*CCL28* and *CFD*) and lower expression of others (*CLU*, *LEAP2*, *PIGR* and *TFF3*) (Figure 4B).

**Figure 5:**
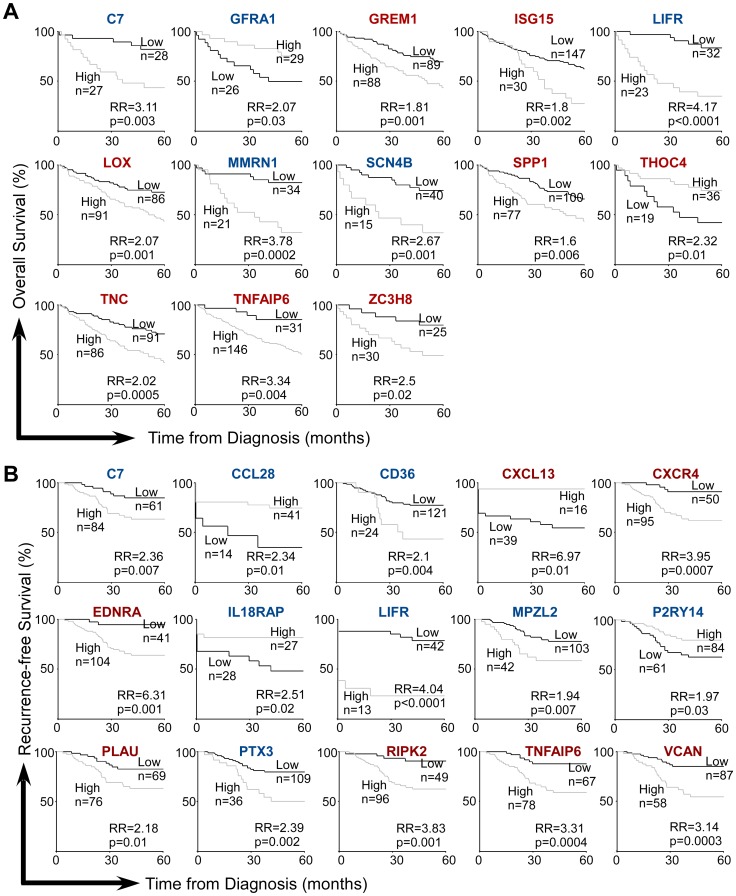
Inflammatory genes predicting overall (A) and recurrence-free (B) survivals in colon cancer. Two datasets from Smith *et al.* [37] were examined for the indicated genes. High, patients with high gene expression. Low, patients with low gene expression. RR, relative risk. Genes are listed in alphabetical order. Red: Elevated iCAMP gene, Blue: Repressed iCAMP gene.

**Figure 6:**
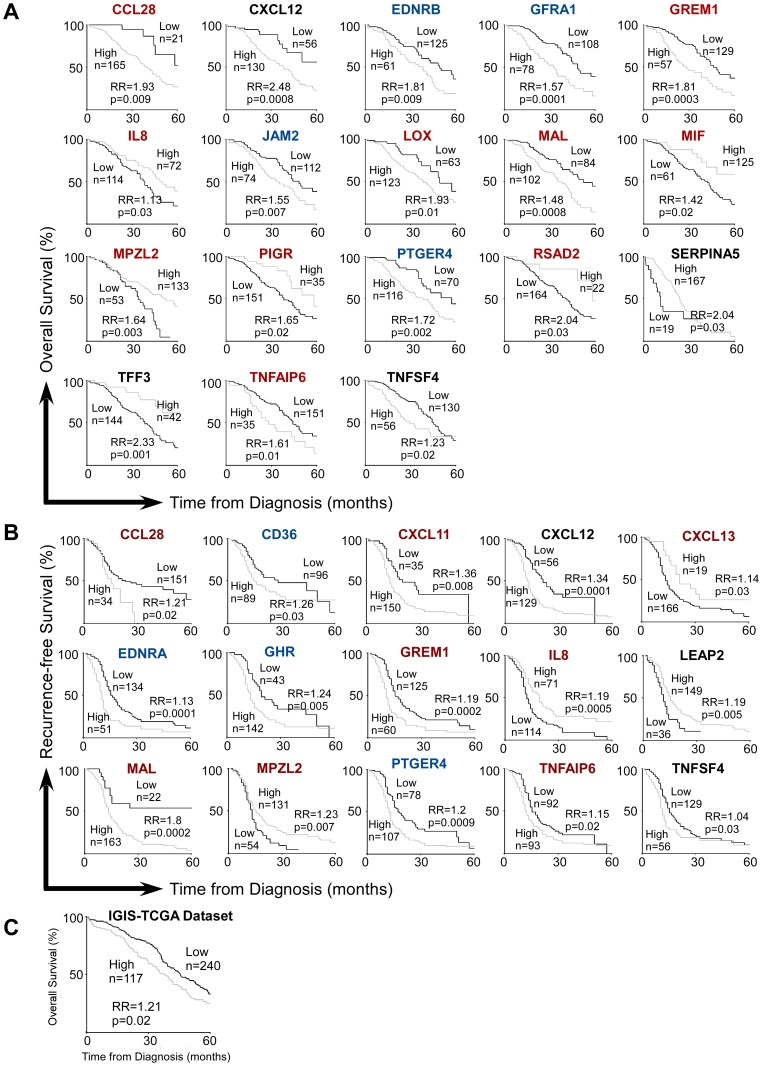
Inflammatory genes predicting patient prognosis in stage IIIC ovarian cancer. A. Overall survival. B. Recurrence-free survival. C. IGIS predicts overall survival in TCGA dataset. Raw gene expression data was obtained from Tothill *et al.* [22] for (A) and (B), and from TCGA for (C). Red: Elevated in ovarian cancer, Blue: Repressed in ovarian cancer, Black: Unchanged or unknown. IGIS score was calculated based on all the 18 genes indicated in (A).

We next determined if iCAMP gene expression level predicts survival, based on raw microarray data for colon cancer [37] and ovarian cancer [22]. Genes which showed different expression levels between survivors and non-survivors at one, three and/or five years were tested by Kaplan-Meier analysis (Figures 5 and 6). In colon cancer, better overall survival was predicted with higher levels of GFRA1 and THOC4, and lower levels of C7, GREM1, ISG15, LIFR, LOX, MMRN1, SCN4B, SPP1, TNC, TNFAIP6 and ZC3H8 (Figure 5A). The dysregulated expression of many iCAMP genes also has significant predictive value for cancer recurrence (Figure 5B).

In stage IIIC ovarian cancer, improved overall survival was associated with higher mRNA levels of IL8, MIF, MPZL2, PIGR, RSAD2, SERPINA5 and TFF3, but lower levels of CCL28, CXCL12, EDNRB, GFRA1, GREM1, JAM2, LOX, MAL, PTGER4, TNFAIP6 and TNFSF4 (Figure 6A). The dysegulation of many of these genes also predicts recurrence-free survival (Figure 6B).

Interestingly, lower expression of a number of genes (GREM1, LOX, TNFAIP6, CD36, and EDNRA) predicted better prognosis in both ovarian and colon cancers. On the other hand, CXCL13 elevation predicted better prognosis in both diseases (Figures 5 and 6).

To determine the prognostic value of iCAMP genes as a group, an inflammatory gene integrated score (IGIS) was devised for ovarian cancer (Figures 6C). IGIS included 18 genes which independently showed significant predictability (p<0.05) by Kaplan-Meier analysis. This score takes into consideration the number of genes within which the patient falls in the high risk group, as well as the risk each of these genes confers. The robustness of IGIS was validated using the independent ovarian cancer TCGA dataset. Based on cutoff values pre-determined from the initial training dataset above [22], stage IIIC patients from the TCGA dataset were categorized as either high or low risk for each gene. Then, for each gene where a TCGA patient shows poor prognosis, relative risk (RR pre-determined by the training dataset) of that gene was added to the patients IGIS score. The TCGA patients eventually were distributed with different IGIS scores based on expression levels of all 18 IGIS genes. We found that IGIS indeed predicted overall survival (RR = 1.21, p = 0.02) (Figures 6C).

### Clinical significance of KPNA2 in colon adenocarcinoma and head-neck squamous cell carcinomas

We next focused on KPNA2 in colon adenocarcinoma and head/neck squamous cell carcinoma, since KPNA2 overexpression has not been reported in these two cancer types. KPNA2 is a nuclear/cytoplasmic protein involved in the import of select cytoplasmic proteins into the nucleus. It binds to the nuclear localization sequence (NLS) of its cargo protein and to karyopherin β1, and the whole protein complex translocates across the nuclear envelope through the nuclear pore complex (NPC) [41]. Among KPNA2 clients is the interferon-γ-induced transcription factor IRF-1 [29] and STAT1 [28], both of which are involved in host immune response.

**Figure 7:**
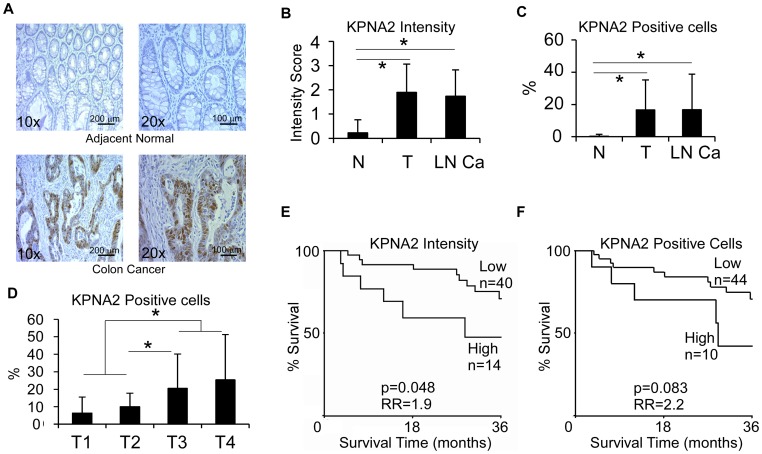
Correlation of KPNA2 expression with colon cancer stage and survival. **A.** Representative images of KPNA2 immunohistochemical staining from normal and malignant colon tissues. **B.** Quantification of KPNA2 staining intensity in normal tissues (N, n = 50), primary tumor (T, n = 55) and lymph node metastasis (LN Ca, n = 15). *p<0.0001. **C.** Frequency of KPNA2 positive cells. *p<0.0001. **D.** The percentage of KPNA2-positive cells correlates with T stage. *p<0.05. **E.** Kaplan-Meier curve showing the overall survival of colon cancer patients with different KPNA2 expression.

We examined the KPNA2 expression with immunohistrochemistry using our in-house tumor microarray which contains 55 primary colon cancer specimens, 15 lymph node metastases and 50 corresponding adjacent normal tissues from patients of various disease stages. We found a drastic increase in KPNA2 expression in primary and lymph node metastatic colon tumors compared to adjacent normal tissues (Figures 7A, 7B and 7C). KPNA2 expression also correlated with the tumor (T) stage, where the percentage of positive cells increased from T1 through T4 (T1: 6.4%, T2: 10%, T3: 20.6% and T4: 25.4%) (Figure 7D). Given that most of the patients were in the T2 (n = 11) and T3 (n = 31) categories, we found significant difference in KPNA2 expression between T2 and T3 (p = 0.017). The difference was also significant between combined T1–T2 and T3–T4 stages (p = 0.003) (Figure 7D). More importantly, patients with KPNA2 intensity score ≥3 displayed worse overall survival than those with KPNA2 intensity ≤2 (Relative risk = 1.9, p = 0.048) (Figure 7E).

**Figure 8:**
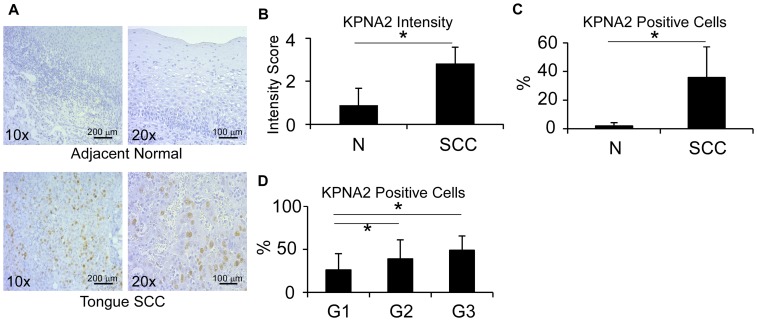
Preferential expression of KPNA2 in head-neck SCC. **A.** Representative images of KPNA2 immunohistochemical staining from normal and malignant tongue tissues. **B.** KPNA2 staining intensity in primary tumor and adjacent normal tissue *p<0.0001. **C.** Frequency of KPNA2 positive cells in primary tumor and adjacent normal tissue (2%). *p<0.0001. **D.** Correlation between KPNA2 expression and tumor grade. *p<0.05.

Similar to colon cancer, oral and laryngeal squamous cell carcinomas displayed elevated levels of KPNA2 compared to independent mouth and larynx normal tissues (Figure 8A, 8B and 8C). Although KPNA2 showed no correlation with the disease stage, it was higher in each of grade 2 (39.2% positive cells) and grade 3 (49% positive cells) carcinomas compared to grade 1 (26.3%). (p(G1 vs. G2)  = 0.03 and p(G1 vs. G3)  = 0.002) (Figure 8D).

### TNFAIP6 is overexpressed in colon adenocarcinoma

**Figure 9:**
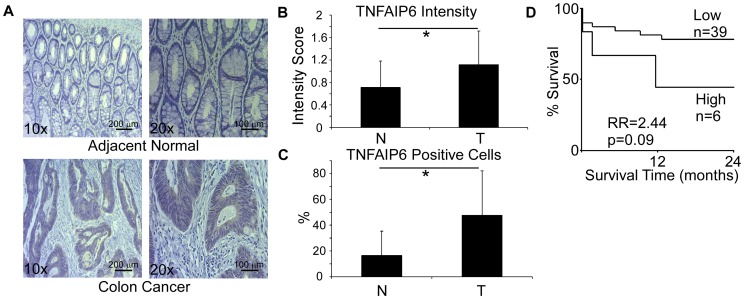
Elevation of TNFAIP6 in colon cancer. **A.** Representative images of TNFAIP6 immunohistochemical staining from normal and malignant colon tissues. **B.** TNFAIP6 staining intensity in primary colon tumor (n = 55) and adjacent normal tissue (n = 50). *p<0.001. **C.** Frequency of TNFAIP6 positive cells in primary tumor (n = 55) and adjacent normal tissue (n = 50). *p<0.0001. **D.** Kaplan-Meier curve showing recurrence-free survival of colon cancer patients with different TNFAIP6 expression, based on the percentage of positive cells.

Tumor necrosis factor alpha-induced protein 6 (TNFAIP6) is a secreted glycoprotein expressed by epithelial cells and leukocytes under normal and inflammatory conditions. Its anti-inflammatory function is well established in different inflammatory conditions such as osteoarthritis, and it is detectable in serum samples from patients with autoimmune disorders [42]. Recently, transcriptional profiling of blood from colorectal patients and normal controls by qRT-PCR identified TNFAIP6 as a biomarker for colorectal cancer [43]. This study shows that TNFAIP6 mRNA is elevated in peripheral blood cells of colorectal cancer patients. Here, we investigated the protein levels of TNFAIP6 in colon cancer cells and adjacent normal epithelium. Immunohistochemical staining of 55 colon cancer specimens and 50 adjacent normal tissues revealed an increased expression in cancer on a per cell basis, as well as an increase in the frequency of TNFAIP6-expressing cells (Figures 9A, B and C). TNFAIP6 protein levels did not predict overall patient survival (data not shown). As for disease recurrence, we found a trend towards worse recurrence-free survival in highly expressing patients (Figure 9D), without reaching statistical significance (RR = 2.44, p = 0.09).

## Discussion

This study was designed to conceptually and experimentally address a novel hypothesis that cancers, irrespective of their etiology, harbor shared iCAMPs to evade immune surveillance and to highjack host immunity for promoting onco-inflammatory growth and metastasis. An unbiased and comprehensive data-mining strategy was undertaken to address this hypothesis and to mine common molecular expression patterns in a large number of patients from many independent studies to ensure the consistency of our findings. The expression of selective genes was confirmed by tissue microarrays. Although the biological significance of our finding awaits further studies, it is clear that the iCAMP not only exists but also shows significant clinical relevance. The core iCAMP gene set reproduces many well-established genes that are aberrantly expressed in cancer tissue, such as VCAN and KPNA2. It also uncovers a number of new consistently dysregulated genes that have not been studied in the context of anti-cancer immunity, such as BST2, LEAP2, TNFAIP6, TNFSF4 and KPNA2 (Table 1).

**Table 1:** Selected iCAMP genes consistently elevated or repressed in cancer and their clinical significance.

Elevated Genes	Background Information	Current Findings
**EDNRA**	Up-regulated in colon cancer [63]	Higher levels confer worse survival in colon and ovarian cancers
**SPP1**	Elevated in multiple types of carcinoma [64]	Elevated with higher stages of ovarian cancer – Predicts worse overall survival in colon cancer
**KPNA2**	Correlates with worse disease and survival [24], [26], [65]	Up-regulated in several cancer types – Correlates with poor prognosis in ovarian, head/neck and colon cancer
**BST2**	Poorly studied in the context of cancer	Elevated in gastric, pancreatic, oral, breast, colon and lung cancers by mRNA and/or IHC.
**TNC**	Elevated in multiple cancers – Induces focal adhesion [66]	Over-expression confers poorer survival in colon cancer.
**TNFAIP6**	Induces the stability of the ECM and promote cell migration – Elevated mRNA in peripheral blood of colon cancer patients [43]	Up-regulated in metastatic ovarian cancer – Elevated with higher T stage – Predicts worse survival in ovarian and colon cancers
**TNFSF4**	Dendritic cell co-stimulatory molecule	Confers worse survival in ovarian cancer – Positively correlates with grade, nodal stage and tumor size in colon cancer
**LOX**	Hypoxia-induced mediator of metastasis – Recruits CD11b+ cells to metastatic niche facilitating malignant cell seeding [67]	Up-regulated in 6 of 7 cancer types – Higher levels confer worse survival in colon and ovarian cancers
**VCAN**	Upregulated in tumors of the breast, ovary, colon and prostate – Confers poor prognosis in endometrial cancer patients [68]	Higher levels predict worse survival in colon and ovarian cancers

Repressed Genes	Background Information	Current Findings
**LEAP2**	Expressed in gastro-intestinal epithelial tissue – Upregulated in response to *Salmonella enterica* and TLR ligands – Role in tumorigenesis unknown [69], [70]	Down-regulated in multiple cancer types – Lower expression predicts worse survival in ovarian cancer
**MPZL2**	Cellular localization and homology to myelin suggest its involvement in cellular attachment – Unclear roles in cancer	Decreased in higher grade ovarian cancer – Lower with higher stage colon cancer – Lower expression predicts worse survival in ovarian cancer – Lower expression predicts worse survival in colon cancer
**PIGR**	Decreased in non-small cell lung cancer [71], [72] – Elevation predicts earlier recurrence of human hepatoma – Promotes metastasis in mouse models [73]	Down-regulated with higher stage ovarian cancer – Lower expression predicts worse survival in ovarian cancer
**LIFR**	IL6 type cytokine receptor – Suppressed in colorectal and liver cancers [74], [75]	Down-regulated on the mRNA level in 6 of the 7 cancers and on the protein level in all 7 cancer types
**PTX3**	A glycoprotein secreted by antigen presenting cells, endothelial cells and fibroblasts in response to inflammatory stimuli [76]	Lower expression predicts better survival in colon cancer – Lower expression in grade 1 compared to grade 3 ovarian cancer
**SERPINA5**	Inhibitor of urokinase-type plasminogen activator (UPA) – Downregulation in advanced-stage ovarian cancer [77]	Lower expression confers worse survival in ovarian cancer – Higher in patients with metastatic disease
**CCL28**	Secreted by ovarian cancer cells in response to hypoxia to mediate Treg recruitment – Higher expression predicts worse survival in ovarian cancer [78]	Increased CCL28 in ovarian cancer with nodal metastasis – Higher levels predict worse overall and recurrence-free survival – Consistently repressed in other cancer including colon, breast, prostate, gastric and pancreatic cancers – lower expression in colon cancer predicts worse survival

The existence of iCAMP highlighted the importance of “onco-inflammation” as one of the common drivers for oncogenesis [44]. The consistent dysregulation of these genes across cancers of different histological origins suggests similar set of “rules” all cancers have to follow in order to sabotage the host immune response and engage it in growth and invasion. Remarkably, these iCAMP genes can potentially be harnessed as biomarkers for cancer aggressiveness, as we found striking predictability of these genes for disease recurrence and patient survival in a number of cancers. The fact that these molecules show consistent aberrant expression and prognostic significance also suggests that they could potentially be therapeutic targets for multiple cancers.

Data-mining is helpful not only in identifying biomarkers and potential therapeutic targets, but also to uncover the underlying biology of human cancer. As an example, molecular profiling of colon cancer revealed higher levels of T cell homing and adhesion molecules to confer more favorable prognosis [45]. In our study, the iCAMP gene set is enriched for genes in positive regulation of lymphocyte apoptosis, which could be a defense mechanism for cancer cells to decrease the pressure of anti-tumor immunity. CXC chemokines are also enriched among the over-expressed genes. These chemokines have been reported to play different roles, ranging from recruiting inflammatory cells to aid cancer invasion to dampening the positive signaling in antitumor immunity.

As for down-regulated genes, they were mostly enriched for immune effector molecules such as granzyme A, Mal and NKG2D (Figure S2B). In fact, NKG2D, which confers activation and anti-tumor immunity in T cells and NK cells, was shown to be shut down in esophageal cancer [46]. Moreover, ligands of this receptor were down-regulated in colorectal cancer [47], which provides a mechanism for evading immune-surveillance. Similarly, granzyme A is part of the cytotoxic T cell and eosinophil arsenal for tumoricidal activity [48]. Mal, which was first described in myelin and lymphocytes was later reported to function in apical transport in epithelial cells, thus, its disruption could lead to disorganized polarity [49]. This protein was down-regulated in multiple types of cancer, including breast and head and neck cancers [49], [50].

The concept of iCAMP is analogous to pathogen-associated molecular patterns (PAMPs) that was postulated more than two decades ago [51]. Importantly, not all up/down-regulated genes contribute to the invasiveness of the cancers studied. The tumor microenvironment consists of a complex interaction among cancer, stromal, endothelial, and immune cells. Any given change in gene expression could be an active process initiated by cancer cells and eventually promote invasiveness, or alternatively, be a reaction by other cells of the immune system. This is best illustrated by some up-regulated genes whose elevation predicts better prognosis, and down-regulated genes whose repression correlates with longer survival.

Among the genes that drew our attention was KPNA2, which was increased in expression in 6 of 7 cancer types at the mRNA and the protein levels, with limited expression in normal epithelia. KPNA2 is an embryonic antigen, shown to be expressed in mouse embryonic stem cells [52] and normal human testis [53], [54], and emerges again in a variety of epithelial [23], [24], [25], [26], [27], [55], [56] and non-epithelial [57], [58] cancers. Despite the exceptional correlation of this gene with cancer behavior across multiple cancer types, its function in shaping tumor behavior remains unclear. One study showed KPNA2 to promote invasive phenotypes of human breast cancer cells *in vitro* [59], but the mechanism underlying this observation was not investigated. In a cell-free system, KPNA2 was reported to interact with OCT4, a transcription factor involved in maintaining pluripotency and self-renewal in embryonic stem cells [54]. Could KPNA2 expression lead to a significant activity in cancer cells to produce oncogenic inflammatory mediators? This is possible, particularly since KPNA2 does not seem to be a general nuclear importer. The selective transport of some key immunologically important transcription factors such as STAT1 and IRF1 by KPNA2 raises a strong possibility of underlying immunological properties of KPNA2. Thus, the potential dual roles of KPNA2 to regulate inflammation as well as stemness of cells suggests that it could serve as a critical link between chronic inflammation and oncogenesis. This speculation warrants further studies.

Since iCAMP represents a list of multiple gene products, an integrated score or index shall be expected to predict the clinical behavior of cancers, which indeed was proven to be the case for both colon and ovarian cancers. This predictive value of iCAMP as a biomarker awaits further investigation in prospective studies, much like the MamaPrint® [60] and Oncotype DX® [61] in optimizing personalized medical practice. Given the inflammatory function of iCAMP genes, the integrated score could prove effective in identifying high risk patients who would benefit from immunotherapy. In this regard, significant efforts are already undertaken to implement the immune score into the staging of cancer, in conjunction with the tumor (T), lymph node infiltration (N) and distal metastasis (M) parameters [62].

## Supporting Information

Figure S1 **Methodology followed to identify the inflammatory cancer-associated molecular pattern (iCAMP).**
(PDF)

Figure S2 **Enrichment of inflammatory gene ontologies within the cancer-associated molecular pattern.** (A) Up-regulated genes, (B) Down-regulated genes. Using DAVID database, all differentially expressed genes were assigned gene ontology terms based on their known functions in the literature. Immune-related gene ontologies are presented here. The horizontal axis represents the enrichment fold: The proportion of a given functional class within our list relative to its proportion in the whole human genome.
(PDF)

Figure S3 **Distribution of the dysregulated genes in different cellular compartments.** Obtained from WebGestalt gene set analysis toolkit. (http://bioinfo.vanderbilt.edu/webgestalt/).
(PDF)

Figure S4 **Distribution of dysregulated genes, their functional classes and previously established interactions.** Each line represents a direct interaction corroborated by at least one evidence from the literature. Obtained from Ingenuity Pathway Analysis (www.ingenuity.com).
(PDF)

Figure S5 **Immunohistochemical stains of selected genes on representative specimens from the Human Protein Atlas (HPAT). Up-regulated Genes.** A: FN1, STAT1, BST2; B: TNC, COL3A1, ISG15; C: PLA2G7, KRT8, CXCL11. **Down-regulated Genes.** D: UNC13B, C7, CDO1; E: TGFBR3, PITGR4, IL1R2; F: SCNN1B, MST1, LIFR; G: PTX3, AZGP1, GIMAP5; H: VSIG2.
(PDF)

Table S1 **GEO datasets.** Summary of the GEO datasets from which differentially expressed genes were identified. HG-U133_Plus_2] Affymetrix Human Genome U133 Plus 2.0 platform was used in all datasets.
(PDF)

Table S2 **Immune-related genes and their corresponding mRNA ratios (cancer:normal).** Shaded genes are those presented in figure 1. These genes have a Fold Change (FC)>2 in 3–7 cancer types, in addition to some genes whose FC values are slightly less than 2 in more than 3 cancer types.
(PDF)

Table S3 **Protein expression of iCAMP genes.** Protein expression changes (Interpreted from the Human Protein Atlas immunohistochemical stains) of the up-regulated and down-regulated genes which showed mRNA differential expression with a fold change FC>2 in 3–7 cancer types. ↑, elevated. ↓, repressed. ↔, no change. NA, Not available.
(PDF)

Table S4 **The number of Oncomine datasets showing elevation or repression of each gene in ovarian cancer.** U, up. D, down. X, no change.
(PDF)
