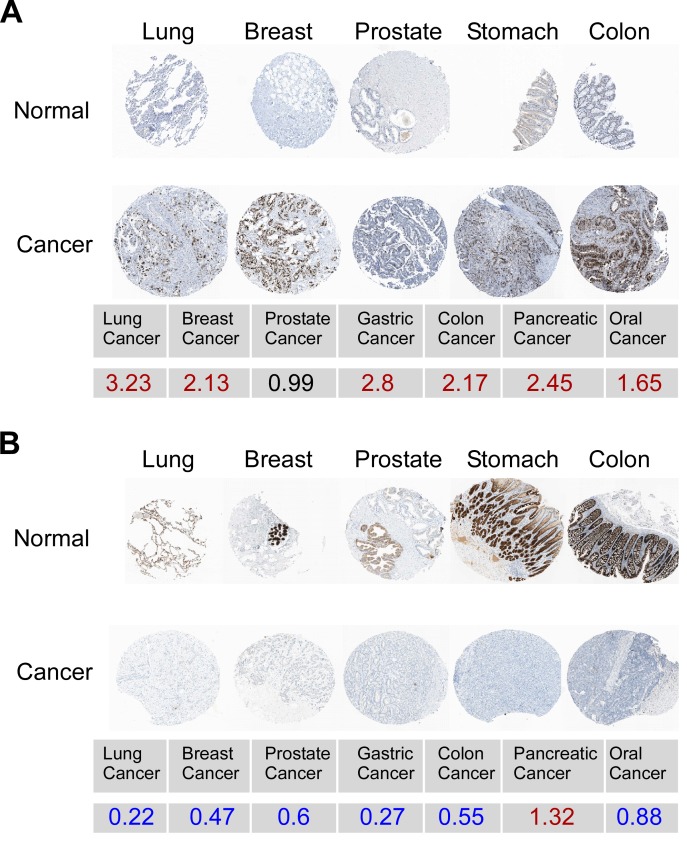# Correction: Molecular Profiling of Multiple Human Cancers Defines an Inflammatory Cancer-Associated Molecular Pattern and Uncovers KPNA2 as a Uniform Poor Prognostic Cancer Marker

**DOI:** 10.1371/annotation/a8159928-d073-4b5b-8a50-c68c95b78681

**Published:** 2014-01-21

**Authors:** Saleh M. Rachidi, Tingting Qin, Shaoli Sun, W. Jim Zheng, Zihai Li

In Figure 2B, some of the organs are mislabeled. Please see the corrected Figure 2 here: 

**Figure:**