# Supplementary figures and images for: Molecular Profiling of Multiple Human Cancers Defines an Inflammatory Cancer-Associated Molecular Pattern and Uncovers KPNA2 as a Uniform Poor Prognostic Cancer Marker
Source: PLoS One. 2013 Mar 25;8(3):e57911. doi: 10.1371/journal.pone.0057911 (PMC3607594; doi:10.1371/journal.pone.0057911)

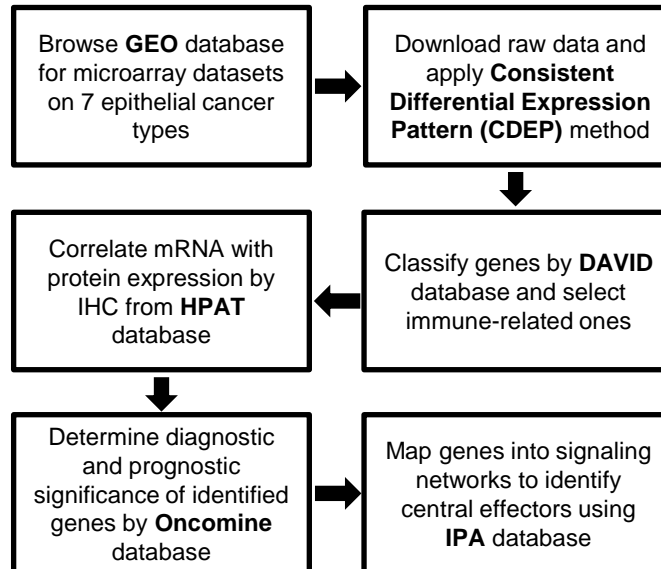

Supplement: Figure S1 — Methodology followed to identify the inflammatory cancer-associated molecular pattern (iCAMP). (PDF) [file pone.0057911.s001.pdf]

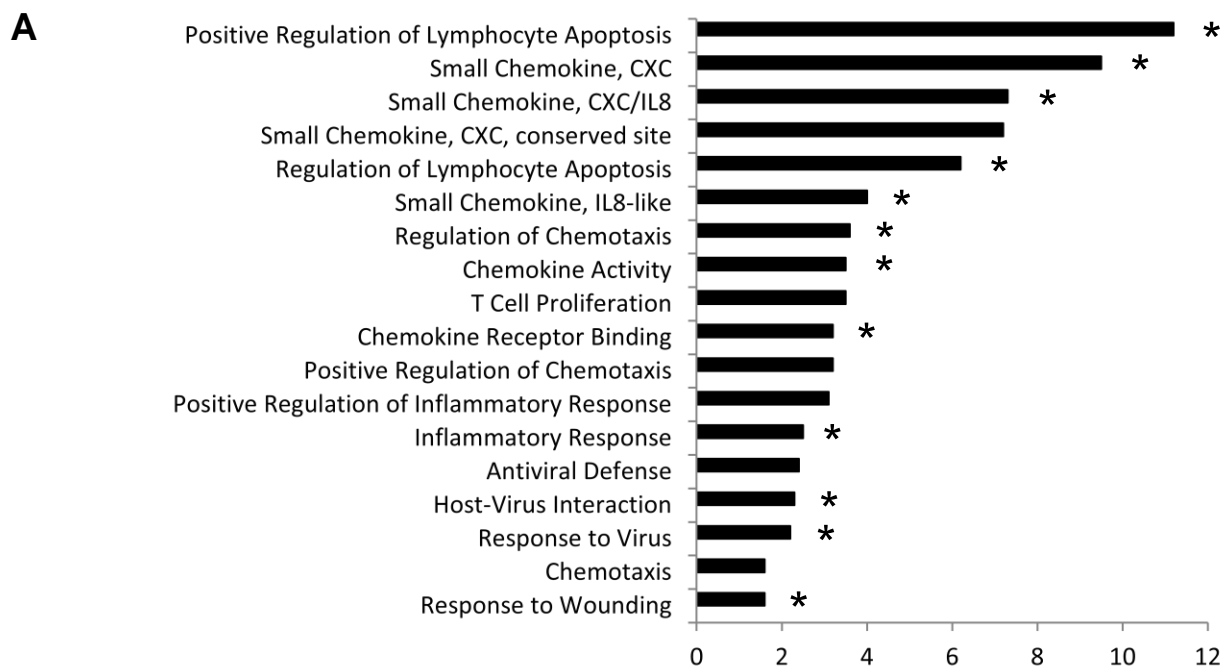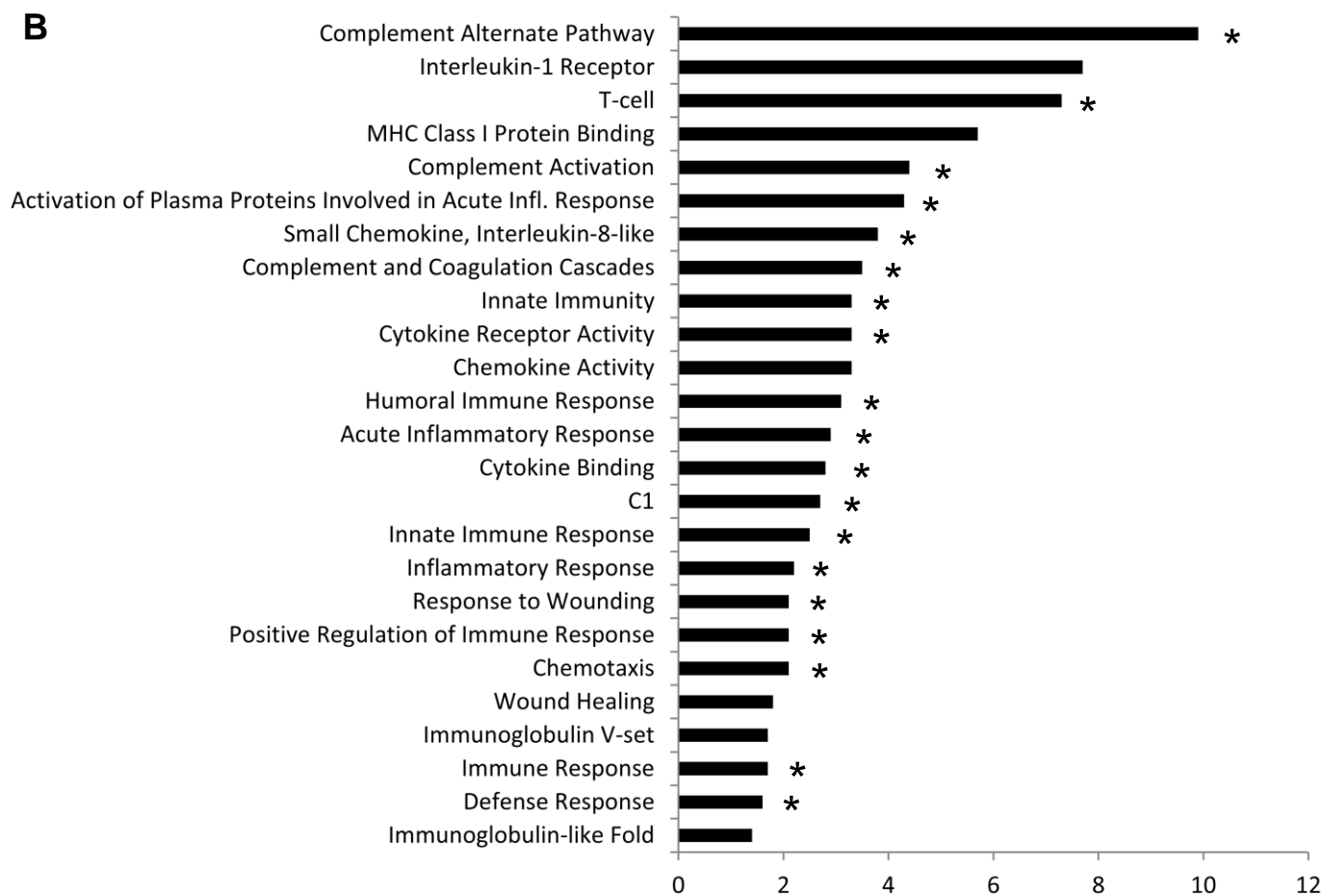

Supplement: Figure S2 — Enrichment of inflammatory gene ontologies within the cancer-associated molecular pattern. (A) Up-regulated genes, (B) Down-regulated genes. Using DAVID database, all differentially expressed genes were assigned gene ontology terms based on their known functions in the literature. Immune-related gene ontologies are presented here. The horizontal axis represents the enrichment fold: The proportion of a given functional class within our list relative to its proportion in the whole human genome. (PDF) [file pone.0057911.s002.pdf]

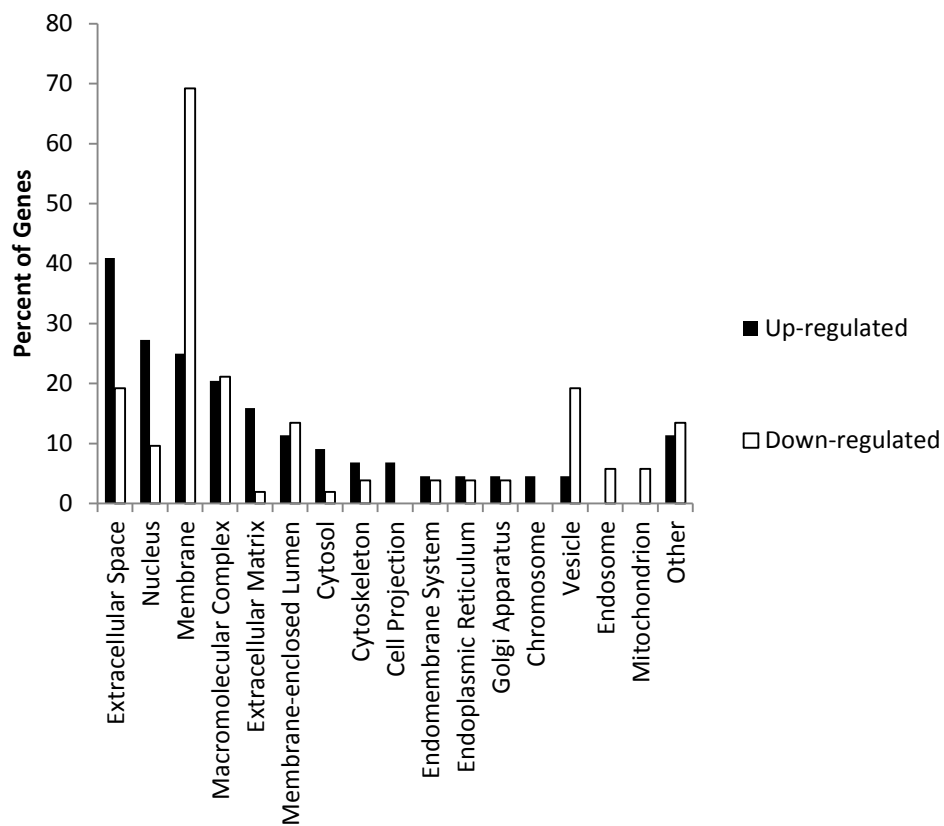

Supplement: Figure S3 — Distribution of the dysregulated genes in different cellular compartments. Obtained from WebGestalt gene set analysis toolkit. (http://bioinfo.vanderbilt.edu/webgestalt/). (PDF) [file pone.0057911.s003.pdf]

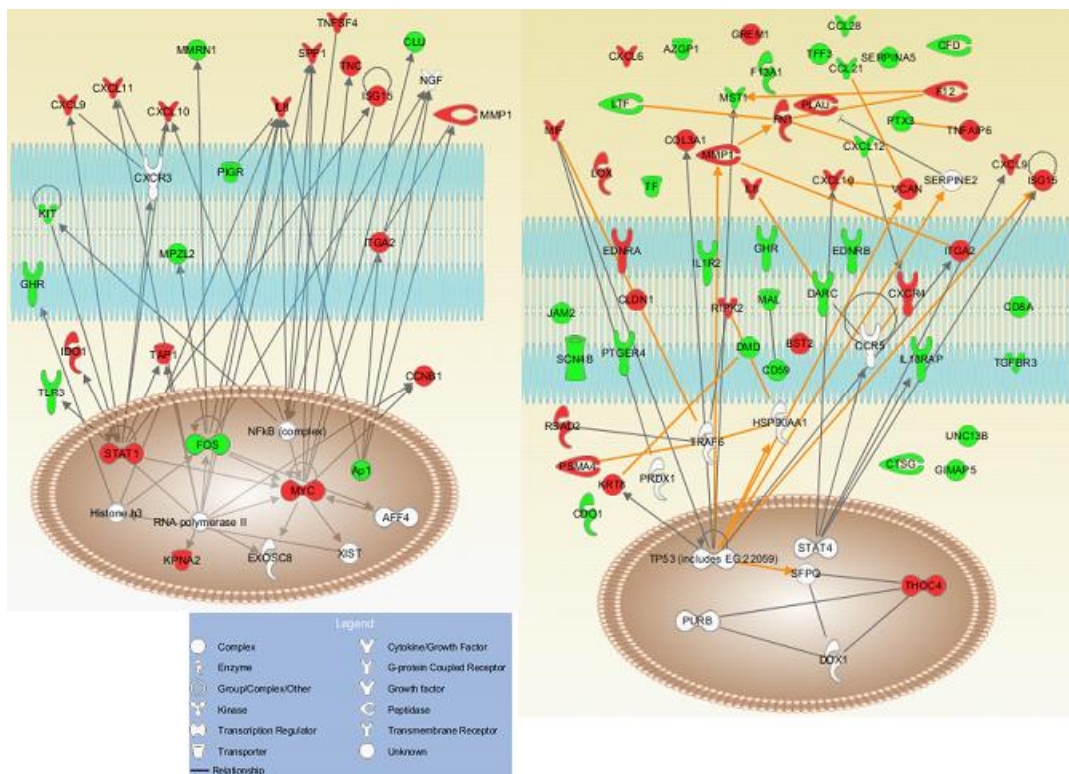

Supplement: Figure S4 — Distribution of dysregulated genes, their functional classes and previously established interactions. Each line represents a direct interaction corroborated by at least one evidence from the literature. Obtained from Ingenuity Pathway Analysis (www.ingenuity.com). (PDF) [file pone.0057911.s004.pdf]

**Figure S5 A**

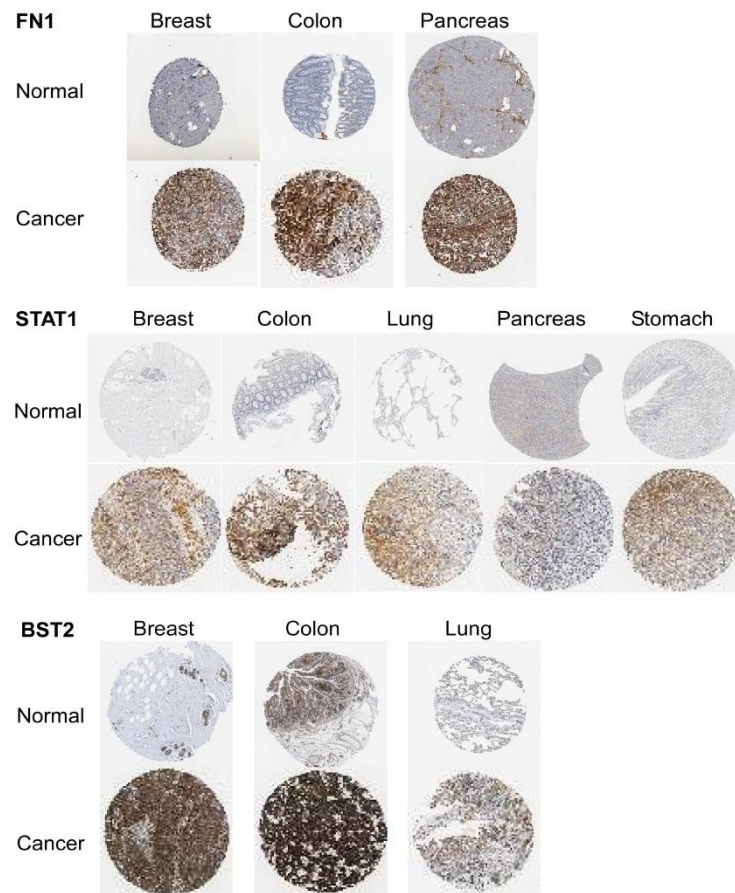

**Figure S5 B**

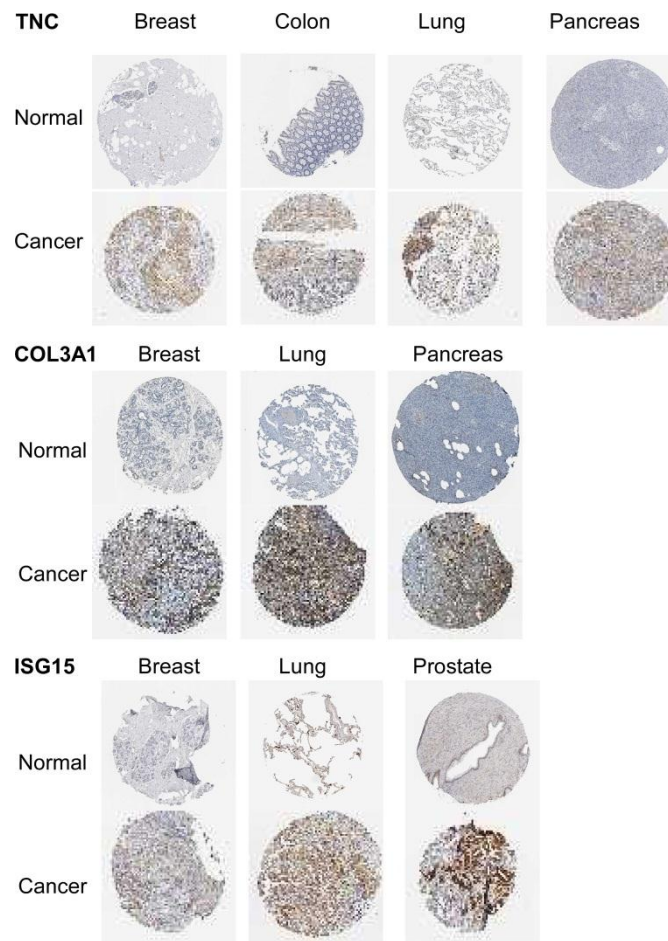

**Figure S5 C**

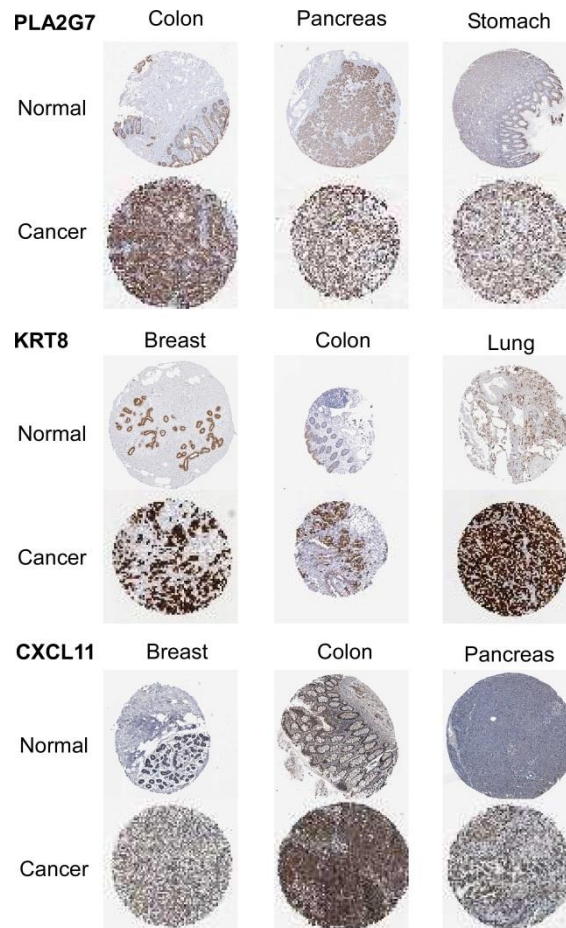

**Figure S5 D**

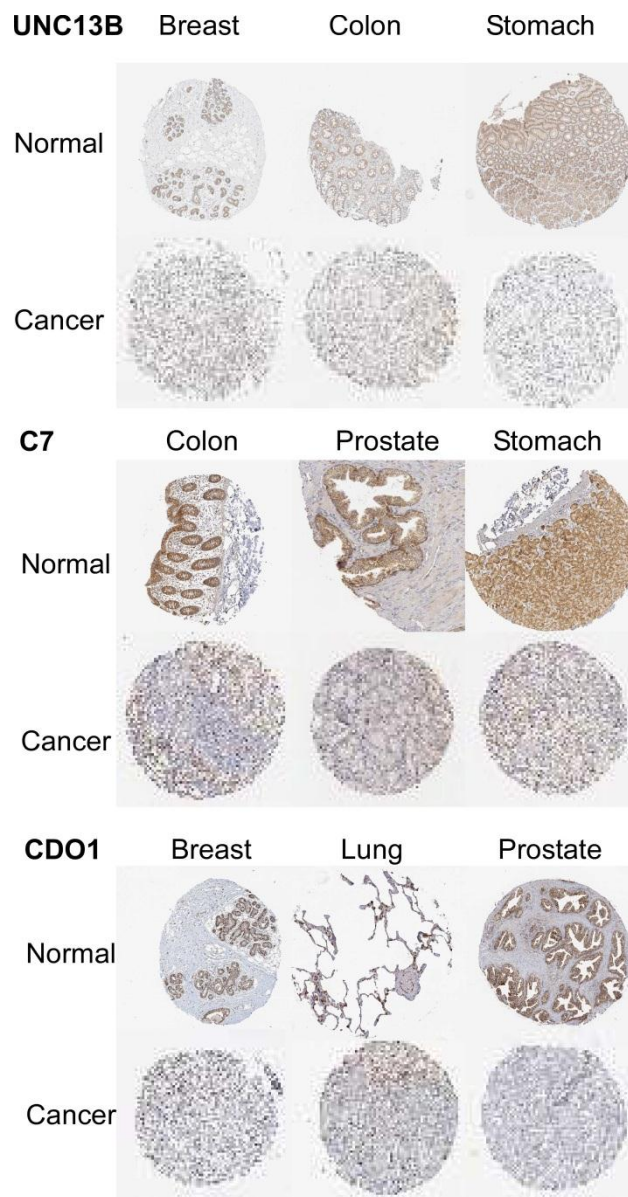

**Figure S5 E**

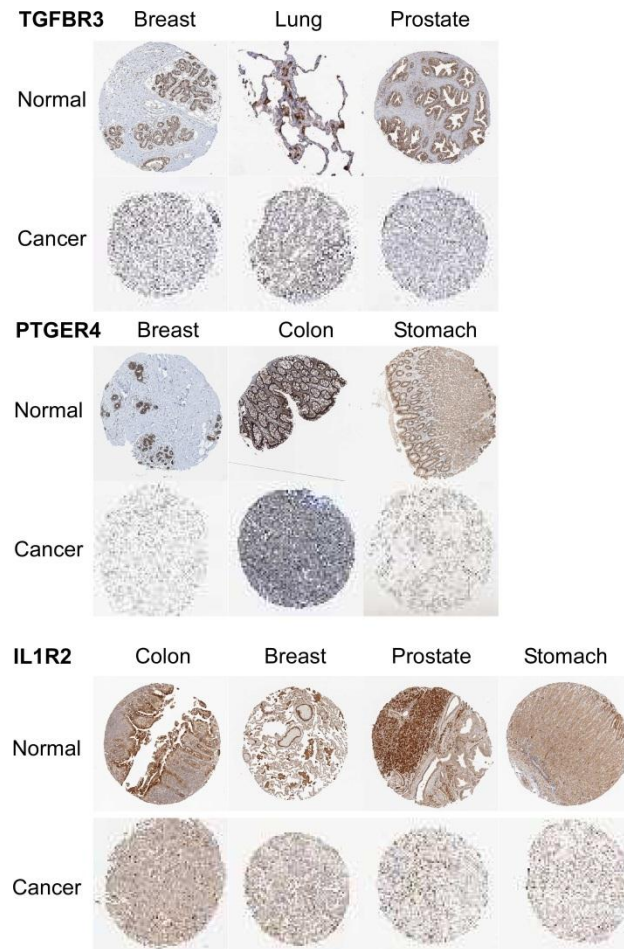

**Figure S5 F**

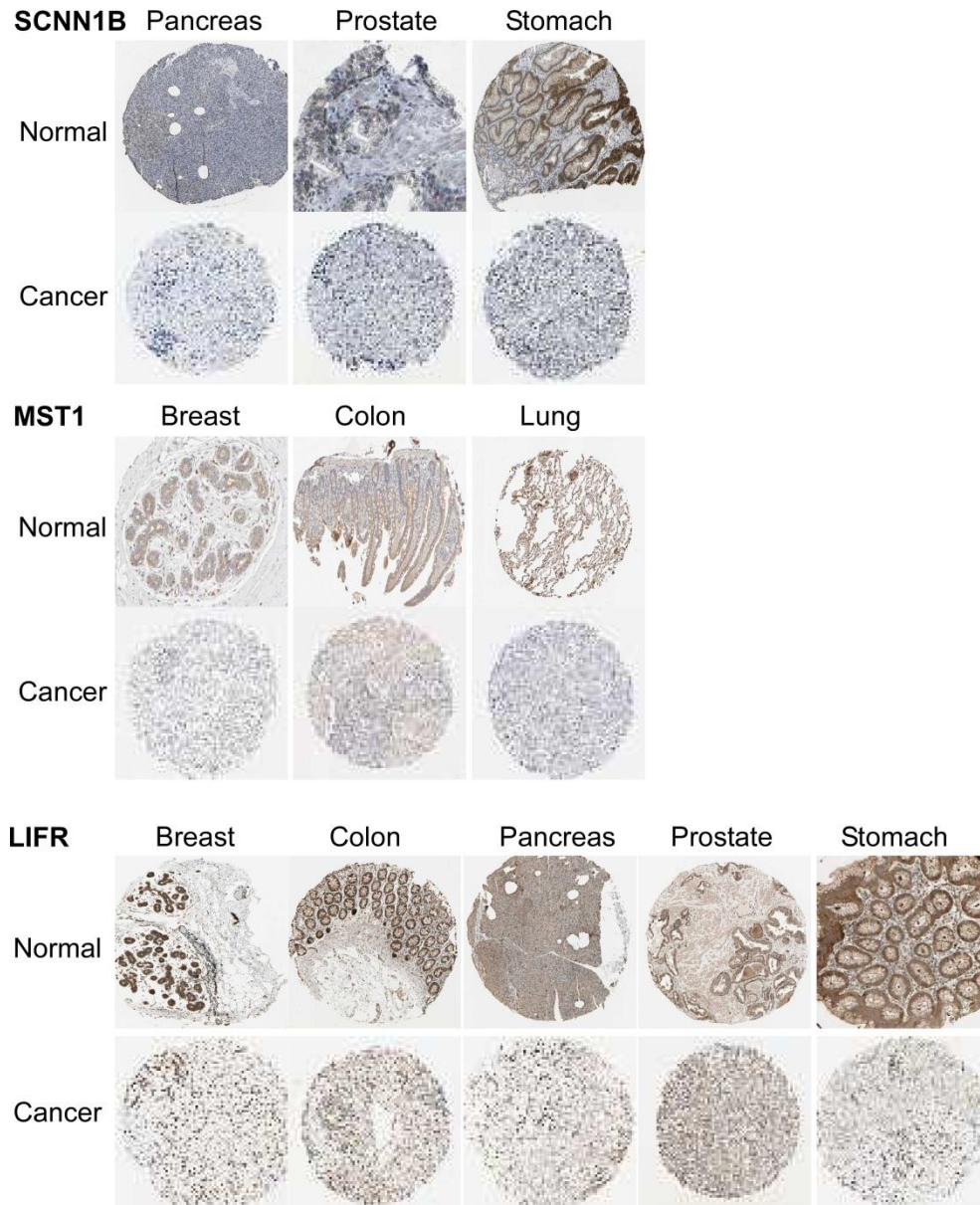

**Figure S5 G**

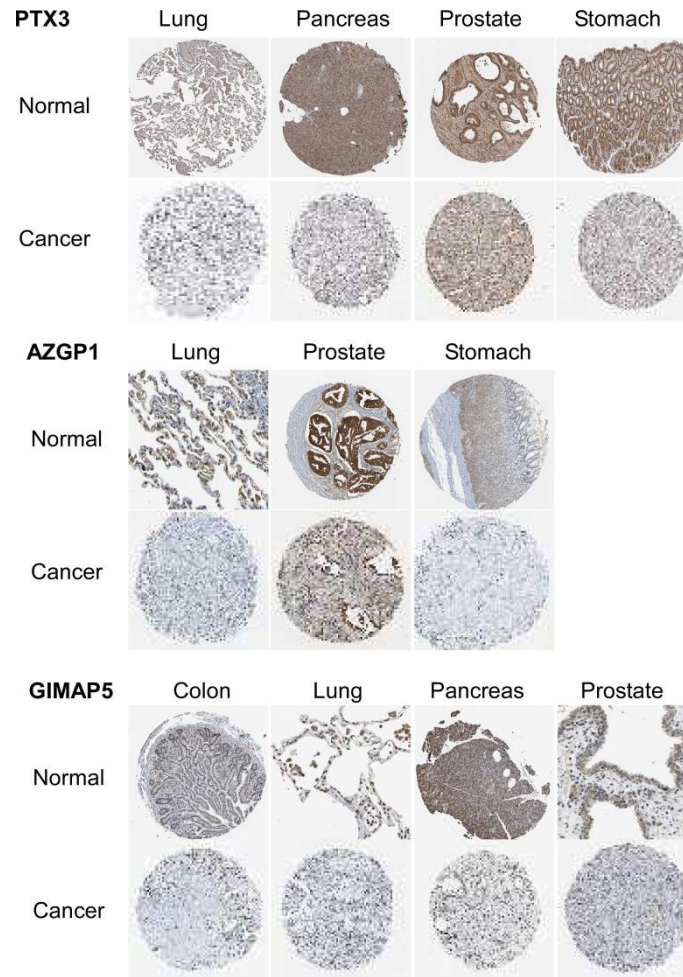

**Figure S5 H**

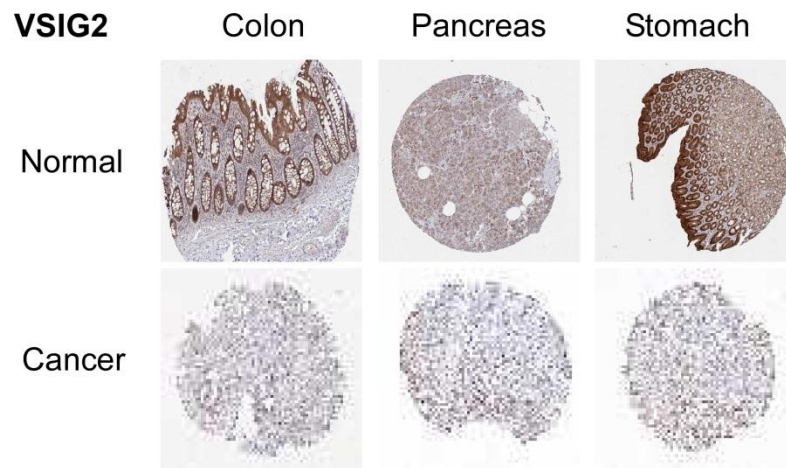

Supplement: Figure S5 — Immunohistochemical stains of selected genes on representative specimens from the Human Protein Atlas (HPAT). Up-regulated Genes. A: FN1, STAT1, BST2; B: TNC, COL3A1, ISG15; C: PLA2G7, KRT8, CXCL11. Down-regulated Genes. D: UNC13B, C7, CDO1; E: TGFBR3, PITGR4, IL1R2; F: SCNN1B, MST1, LIFR; G: PTX3, AZGP1, GIMAP5; H: VSIG2. (PDF) [file pone.0057911.s005.pdf]
