# Supplementary material for: Molecular Profiling of Multiple Human Cancers Defines an Inflammatory Cancer-Associated Molecular Pattern and Uncovers KPNA2 as a Uniform Poor Prognostic Cancer Marker
Source: PLoS One. 2013 Mar 25;8(3):e57911. doi: 10.1371/journal.pone.0057911 (PMC3607594; doi:10.1371/journal.pone.0057911)
Supplement: Table S1 — GEO datasets. Summary of the GEO datasets from which differentially expressed genes were identified. HG-U133_Plus_2] Affymetrix Human Genome U133 Plus 2.0 platform was used in all datasets. (PDF) [file pone.0057911.s006.pdf]

Cancer Type	GEO ID	No. of samples
**Breast**	GDS2635	20 controls and 10 cases
**Colon**	GSE20916	44 controls and 91 cases
**Gastric**	GSE13911	31 controls and 38 cases
**Lung**	GSE19188	65 controls and 91 cases
**Oral**	GSE9844	12 controls and 26 cases
**Pancreatic**	GSE15471	36 controls and 36 cases
**Prostate**	GSE17951	13 controls and 32 cases
