# Supplementary material for: Molecular Profiling of Multiple Human Cancers Defines an Inflammatory Cancer-Associated Molecular Pattern and Uncovers KPNA2 as a Uniform Poor Prognostic Cancer Marker
Source: PLoS One. 2013 Mar 25;8(3):e57911. doi: 10.1371/journal.pone.0057911 (PMC3607594; doi:10.1371/journal.pone.0057911)
Supplement: Table S2 — Immune-related genes and their corresponding mRNA ratios (cancer:normal). Shaded genes are those presented in figure 1. These genes have a Fold Change (FC)>2 in 3–7 cancer types, in addition to some genes whose FC values are slightly less than 2 in more than 3 cancer types. (PDF) [file pone.0057911.s007.pdf]

Gene ID	Breast	Colon	Lung	Oral	Pancreatic	Prostate	Gastric
**4069**	3.61692	1	0.51238	0.77199	3.3910962	1.9439	0.73502
**2335**	5.73679	0.94457	0.82662	1.70913	10.234543	0.66112	3.13608
**6696**	1.96551	3.62093	8.49303	1.8327	5.8898119	0.49221	4.66459
**5054**	1.11181	1.98692	0.72937	1.65695	5.4409194	0.73005	2.05328
**7534**	1.49385	1.52104	1.64373	1.32697	1.8924823	1.55557	1.57024
**5901**	1.27362	1.90409	1.25789	1.34704	1.5288425	1.15039	1.84003
**1508**	1.25729	1.31501	1.08885	1.3307	3.3636268	0.39308	2.6237
**6772**	1.90299	1.48803	1.70407	2.00986	2.2869013	1.3894	2.34802
**3838**	2.13289	2.16665	3.23172	1.64886	2.4455145	0.99057	2.83957
**5688**	1.31474	2.39208	1.46607	1.80872	1.4631167	1.0015	2.00303
**5702**	1.65818	2.45651	1.21669	1.16969	1.2023603	1.80023	1.80444
**3491**	0.50432	0.97107	0.28471	1.78708	2.6626292	1.07599	1.09151
**5691**	1.48096	1.7843	1.61686	1.55726	1.6300198	1.6504	1.73588
**3875**	0.3724	1.68335	1.1773	1.74389	1.8961308	8.39813	1.70018
**684**	1.65554	1.11919	0.85269	4.46081	4.0193818	0.25388	3.71834
**3371**	0.57095	2.26511	0.92628	2.78899	2.484036	0.69351	1.56961
**7157**	1.0444	2.88552	1.35557	1.00919	1.3843384	1.98395	1.8822
**1281**	5.1506	1.58642	3.68614	1.82779	9.3066508	0.74927	1.85687
**4599**	1.93039	1.18069	0.71031	3.36527	4.7574982	0.27189	1.1545
**6890**	1.42925	1.1802	0.98901	2.09923	2.9519228	0.58874	2.28307
**4609**	0.7717	4.67809	0.8215	1.8057	0.8338761	7.21226	2.26567
**8772**	1.87366	0.89591	1.58519	2.31059	1.696684	0.97415	1.316
**102**	0.9781	1.15456	0.9186	1.22276	2.2394022	2.98827	1.4648
**6921**	1.29811	1.42674	1.36673	1.37363	1.4787337	1.12408	1.76736
**3576**	1.20461	18.219	0.52177	5.62029	11.884072	0.59821	10.5154
**713**	1.60115	1.98469	0.34801	1.32095	5.7627885	0.16913	1.78201
**1289**	6.32842	2.51775	3.14415	2.69926	23.732603	0.47771	3.41488
**5685**	1.6089	1.14663	1.44169	1.59773	1.4799699	1.82315	1.53675
**5320**	1.75998	0.53504	0.58795	0.60261	1.9612276	1.18309	6.3504
**7412**	2.60212	0.8389	0.9212	0.57332	2.1367279	0.37647	1.91481
**10379**	2.73477	1.98121	0.87889	1.76406	2.7810469	1.08498	1.24941
**4283**	3.47035	1.86751	2.40113	1.48314	4.5364785	1.38034	8.1601
**4015**	1.74658	1.35669	1.91329	1.95953	4.8188131	0.38265	1.58773
**2152**	0.37519	1.54709	0.48962	1.93598	2.6740183	1.5701	1.07024
**1909**	2.37067	2.84569	0.40513	1.15134	10.975621	0.87896	2.09764
**4312**	2.01346	44.9918	15.8782	264.704	13.973556	0.20448	2.7407
**3627**	9.133	5.45456	2.0562	4.59187	5.1300199	2.71448	6.97681
**1462**	7.22994	2.44197	1.83513	1.26208	23.04027	0.2751	2.49583
**5653**	0.98345	2.59978	1.37408	1.31937	1.9499583	0.99947	1.71036
**3932**	1.71413	1.33859	0.83005	0.87706	2.377017	0.36309	1.47513

**1058**	1.23368	1.89329	5.17739	1.67783	1.2339801	2.61448	2.2009
**3673**	0.39307	2.4689	1.23205	1.56435	6.5957574	0.3552	2.68581
**3556**	0.77321	1.44873	1.1802	1.68785	2.8473226	1.15082	1.4049
**10563**	1.24234	0.4399	19.7912	5.19228	2.793004	0.13212	1.50571
**6364**	1	1.76723	0.52489	7.08309	7.5482486	0.32972	2.48948
**5328**	1.53936	4.44615	2.37221	2.68973	9.693405	0.51891	5.03779
**9636**	5.02837	1.53821	1.59004	8.26314	4.7379245	1.3371	3.27213
**2161**	2.61026	1.69709	3.83549	1.03882	1.0509984	1.12315	2.86718
**624**	2.31689	0.68613	1.10806	1.00356	1.3637886	0.06317	1.21851
**136**	0.84739	3.53753	1.25418	1.73523	1.6124946	0.2219	2.30109
**2960**	1.39468	0.9501	1.45898	1.45359	1.5880803	1.43381	1.37685
**7130**	2.50076	2.71134	1.08756	2.13865	9.2679512	0.11254	4.26063
**7941**	1.38434	1.67201	1.4839	1.38943	5.2780282	8.60872	5.0903
**4277**	2.12796	1.86102	0.93814	2.21246	2.9734547	0.10423	2.98207
**6372**	1.10648	4.5771	1.72814	2.64489	5.6502936	0.12774	2.62743
**23643**	1.23034	1.45991	0.9411	1.27706	6.1181561	0.29851	0.99899
**2151**	1.19873	2.19331	1.34175	1.12476	2.8809389	1.38424	1.43402
**7292**	1.03285	1.42132	2.69285	1.46646	4.9154388	1.85613	2.29923
**3665**	2.36098	0.97747	1.12177	2.1551	1.7941195	0.34685	1.54119
**581**	1.00525	2.76236	1.22147	1.03773	1.506503	1.48205	1.45374
**1981**	0.93852	1.6629	1.72644	1.30793	1.2123815	1.82849	1.43344
**5693**	0.9824	1.54503	1.95328	1.94309	1.1715474	1.56501	1.65011
**3856**	0.55223	1.21888	1.73168	1.05204	2.427004	11.3201	1.40319
**5696**	1.37344	1.53807	0.89263	1.52874	4.210184	0.87849	1.63588
**1490**	1.12146	1.77001	0.42829	1.24348	2.9079197	1.48374	1.12681
**7852**	2.05184	1.2642	1.16606	1.16324	9.9693484	0.49674	1.4294
**8767**	1.08357	2.23047	0.86447	1.47509	1.5959565	2.05942	2.04001
**719**	1.85284	1.08353	0.53651	1.2574	2.825292	0.47772	1.51395
**6362**	1.92175	1.3003	0.47038	0.90723	5.7094439	1.36852	3.7281
**3620**	1.79798	2.06476	0.5603	2.54041	1.0859329	0.55688	4.55249
**8815**	1.22243	2.52107	1.58826	1.33676	1.2207466	1.47363	2.09056
**6373**	2.32056	7.82168	1.30525	3.10303	0.4817058	3.40645	4.44536
**7422**	1.29473	2.21955	1.50512	1.43822	1.3285691	1.17596	1.19184
**6091**	1.54469	1.33525	1.13777	0.78063	4.8713797	0.56703	1.99922
**91543**	2.09105	0.44148	0.4761	5.72369	4.0932553	1.36872	0.84566
**891**	2.68508	2.39919	10.7691	1.8171	2.1397106	2.87999	4.32008
**4282**	1.94882	3.10264	3.35993	1.41285	1.4938258	2.04421	2.31046
**9076**	1.08282	8.80746	1.51339	2.94299	3.6706462	0.35518	5.35923
**26585**	1.34	2.6187	26.8908	3.59127	13.175795	0.0936	2.99664
**79682**	2.78775	1.66628	3.26711	1.05459	1.5411834	2.0645	1.64118
**50507**	1.89667	1.32794	1.82021	1.36375	4.3928029	0.62172	1.24337

**64332**	0.71824	1.58061	0.27706	1.17722	2.2288136	1.67516	1.32346
**84524**	1.19424	1.64547	3.13782	1.22555	0.7093986	2.18414	2.12981
**10189**	1.01165	3.12054	2.43582	1.66132	1.4899086	2.48394	3.71745
**83872**	2.64679	1.11463	0.31227	0.60294	6.1961296	0.89264	2.19911
**6352**	1.53984	0.40687	0.62609	0.9309	2.3175505	0.43545	0.9586
**116842**	1.11498	0.21016	0.82572	0.51103	0.5068005	0.52526	0.42708
**64283**	0.507	1.28728	0.72636	1.06669	0.984887	0.40105	0.52868
**83593**	0.72402	0.71529	0.68505	0.72597	1.5004038	0.4406	0.98142
**3603**	1.14888	0.79869	0.67802	0.63015	1.0471718	0.33393	0.927
**3426**	0.44937	4.0355	0.41243	0.80785	3.5074875	0.15847	1.0963
**22914**	1.57331	0.49744	0.48485	0.70353	1.179566	0.52831	0.81779
**5894**	0.67873	0.79947	0.66834	0.61989	1.1367907	0.68864	0.87421
**1191**	0.59564	0.83873	0.38317	0.36861	0.8517715	0.27339	0.37395
**1832**	0.32903	0.75872	8.36851	1.02192	0.8139847	2.77282	0.8044
**966**	0.49018	1.17025	0.43227	0.81578	1.7573317	0.4549	0.62161
**1999**	0.35953	0.76263	0.57988	0.7913	2.3286679	1.08371	1.05235
**4057**	0.18903	0.68645	2.38319	0.56261	1.5004552	0.21824	0.0298
**10497**	0.99478	0.79235	0.31789	0.66787	0.9415023	3.20583	0.89584
**133**	0.31736	0.44509	1.35432	1.98051	1.7726124	0.213	1.14978
**730**	0.87699	0.428	0.19127	0.66781	1.2468312	0.21356	0.66151
**3117**	1.50095	0.77073	0.54121	0.48157	2.5952816	1.31986	1.24367
**2162**	2.60635	0.31283	0.39936	0.55397	4.7701053	0.12147	0.28614
**7018**	0.39112	1.00115	1.61416	0.42292	0.7606488	0.16203	1.1052
**6387**	1.172	0.40414	0.3828	0.11954	0.5947299	0.35634	0.70956
**10205**	0.453	0.75932	0.69457	0.74287	2.3704403	0.52412	0.86739
**9936**	0.64746	0.41183	0.18626	0.33482	0.9425846	0.98464	0.48245
**1756**	0.66997	0.82969	0.74291	0.8782	0.4962809	0.64864	0.70098
**1036**	0.72287	0.90552	0.12908	0.40812	1.2703739	0.30815	1.05061
**5284**	0.47438	0.55496	0.21927	0.88112	1.3219235	0.59583	0.26669
**1910**	0.68796	0.88193	0.07245	0.37726	0.9484816	0.11681	0.71677
**51090**	0.92466	0.4586	0.39601	0.77855	1.020117	0.43136	0.58904
**10752**	0.38988	0.54369	1.3181	0.80319	1.1760222	0.32539	0.81552
**6366**	1.4091	0.63757	0.60477	0.62405	1.5329911	0.1424	0.79775
**7033**	0.52756	0.91571	1.5891	0.67277	1.3837534	26.5791	3.49572
**7049**	0.34887	0.83815	0.1265	0.27149	0.9891324	0.14449	0.94471
**4118**	1.51403	0.49513	0.25083	0.01588	0.5950951	0.02136	0.07515
**11326**	1.51601	0.52374	0.15847	0.66016	4.6010359	0.08082	0.88916
**8639**	1.03187	0.48376	0.11401	0.18949	2.0723631	0.34765	0.60246
**5734**	0.47103	0.34261	0.25692	0.47103	0.4061922	1.06302	0.64399
**3587**	1.55798	0.59771	0.42344	0.79451	3.5285589	0.27891	0.84333
**3385**	1.2056	0.81959	0.73767	0.62345	1.0003373	2.04096	0.72714

**5790**	1.07468	0.61531	0.95929	0.93968	1.0821644	0.249	0.85551
**3815**	0.155	0.36516	0.38843	0.58019	2.0068512	0.13285	0.21728
**1439**	1.3869	0.86121	0.3431	0.81008	3.7859793	0.21356	0.6992
**1675**	0.5881	0.3013	0.11909	0.12059	0.7196095	0.05499	0.43743
**7850**	0.94953	0.13128	0.98417	0.29818	3.4537927	0.12417	0.36124
**6338**	1.01883	0.04933	0.23933	0.50081	0.7955584	0.40235	0.24277
**3001**	2.07589	0.28048	0.6678	0.64276	2.9974442	0.51014	0.92705
**2690**	0.48791	0.08584	0.20695	0.38191	1.2505213	1.85	0.30981
**10125**	2.10247	0.54084	0.56033	0.87427	2.8625537	0.37403	1.07813
**22915**	0.95302	0.60176	0.21724	0.54307	1.0437175	0.03331	0.55588
**4485**	0.56036	0.58179	1.00344	0.78461	0.5644146	0.57083	0.84686
**1511**	0.73003	0.54222	0.51217	0.78011	0.75	0.30015	0.86382
**4065**	0.38213	0.89921	0.62305	0.66284	3.8798656	0.3055	1.66081
**2674**	0.37326	0.8384	0.69854	0.72786	0.9365025	0.35637	0.89441
**2157**	1.18962	0.92325	0.22205	0.72685	0.3463977	0.38683	1.03732
**925**	1.90903	0.34237	0.58453	0.57284	1.3739013	0.51263	0.81077
**914**	1.66329	0.81544	0.67759	0.48287	2.8299467	0.30329	0.97356
**3977**	0.52015	0.38496	0.23093	0.60919	0.3587823	2.68628	0.19375
**5806**	0.28687	1.03929	0.22637	1.44384	0.8016419	0.02539	1.04544
**7098**	1.15578	0.27436	0.35966	0.55821	1.3525577	0.34698	0.47903
**948**	0.55276	0.46093	0.22078	0.71452	0.6767064	0.38655	0.45922
**9934**	0.99087	0.5194	0.21704	0.32012	1.1446561	0.31549	0.37896
**8807**	1.67204	0.6757	0.27406	0.67082	0.8868297	0.09174	1.01808
**4915**	0.53391	0.72013	0.91641	1.01832	0.7419478	0.60075	1.04174
**54941**	1.08554	0.32763	0.36171	0.54109	0.9219845	0.88952	0.99176
**5627**	0.50996	0.29063	0.27245	0.59783	3.3769953	0.70564	1.71905
**1755**	0.99487	2.02981	0.11126	0.91975	3.8625662	0.07466	2.12719
**2532**	0.3948	0.46712	0.10833	0.2743	1.6653752	0.04709	0.64379
**2353**	0.20529	0.31301	0.18702	0.92295	1.4305996	2.37127	0.62883
**563**	0.15909	14.7305	0.31325	1.01182	0.2045629	2.93633	0.12729
**2053**	0.56438	0.35264	0.51525	0.6583	0.2211121	6.15137	0.80169
**5168**	1.04903	0.95854	0.5682	0.31836	2.3683088	0.34828	0.4695
**5104**	0.73594	0.93496	1.22127	0.66893	0.7732326	0.21152	0.68129
**3479**	1.52634	0.46968	0.83159	0.62683	2.3916391	0.44821	0.99809
**28755**	1.92105	0.62933	0.91313	0.67466	2.3892851	0.57114	0.93934
**6363**	1.30443	0.55379	1.74129	0.33625	8.0309224	0.23262	1.06749
**7100**	0.99324	0.61435	0.59375	0.80619	2.2115276	0.53193	0.86146
**1672**	0.79118	0.42917	1.57779	0.879	1.3361336	0.06702	0.59997
**2213**	1.41385	0.69011	0.84791	0.70986	5.9519558	0.235	0.94176
**11343**	1.0675	0.69039	0.26569	0.2488	4.0705681	0.41538	0.78483
**3512**	0.36374	0.80197	0.6723	0.85248	2.2110075	0.68299	0.21469

**10346**	0.75817	1.46031	0.35229	0.99839	2.6281481	0.5081	0.94391
**915**	1.54718	0.89941	0.80865	0.65237	1.5063371	0.47979	0.85461
**3075**	1.21831	0.3446	0.36088	0.22802	4.870638	0.08597	0.54171
**55340**	1.03166	0.74999	0.40245	0.48566	1.0828893	0.40973	0.9005
**3108**	0.99173	1.01314	0.29281	0.51645	3.8088002	0.56553	1.06577
**10451**	0.83175	0.92354	0.89262	0.47956	0.5748606	0.24969	0.96015
**57088**	0.84942	0.25724	0.27673	0.48164	1.6306741	0.39737	0.99316
**10894**	1.03156	0.51045	0.11386	0.99086	0.5942257	0.01884	0.66865
**58494**	0.83821	0.35113	0.15047	0.22191	1.7998154	0.20747	0.55413
**114625**	0.58799	0.70558	0.68004	0.71382	1.0618812	3.20318	0.86697
**51554**	0.96189	0.64315	0.13313	1.16492	0.5512988	0.63516	0.19453
**2635**	1.79294	0.48	0.37434	1.05236	3.2673703	0.33088	0.89364
**56477**	0.14909	0.27353	1.12896	1.11927	0.7630203	0.52499	0.55205
**64115**	0.9142	0.54568	0.33178	0.75056	1.0978653	0.10536	0.82169
**133418**	1.02181	0.58116	0.68622	0.59218	4.1547813	4.59527	0.81439
**284340**	1.02776	1.15239	0.20598	0.51538	0.7542855	0.07303	0.06728
**120425**	1.20534	0.63097	0.18835	0.61993	1.8940262	0.31448	0.81452
**23584**	0.59716	0.17854	0.22815	0.60175	1.7039435	1.11454	0.0745
**6330**	0.45913	0.80489	0.12333	0.4452	1.3573718	0.24907	0.9293
