# Supplementary material for: Molecular Profiling of Multiple Human Cancers Defines an Inflammatory Cancer-Associated Molecular Pattern and Uncovers KPNA2 as a Uniform Poor Prognostic Cancer Marker
Source: PLoS One. 2013 Mar 25;8(3):e57911. doi: 10.1371/journal.pone.0057911 (PMC3607594; doi:10.1371/journal.pone.0057911)
Supplement: Table S3 — Protein expression of iCAMP genes. Protein expression changes (Interpreted from the Human Protein Atlas immunohistochemical stains) of the up-regulated and down-regulated genes which showed mRNA differential expression with a fold change FC>2 in 3–7 cancer types. ↑, elevated. ↓, repressed. ↔, no change. NA, Not available. (PDF) [file pone.0057911.s008.pdf]

	Breast	Colon	Lung	Pancreas	Prostate	Stomach
Up-regulated Genes						
FN1	↑	↑	↔	↑	↓	↔
SPP1	↓	↓	↑	↔	↓	↑
STAT1	↑	↑	↑	↑	↓	↑
KPNA2	↑	↑	↑	↑	↑	↑
BST2	↑	↑	↑	↔	↓	↔
TNC	↑	↑	↑	↑	↔	↑
COL3A1	↑	↓	↑	↑	↔	↓
TAP1	↓	↔	↑	↑	↓	↓
MYC	↔	↔	↔	↔	↔	↔
IL8	NA	NA	NA	NA	NA	NA
COL5A1	↓	↓	↓	↓	↔	↓
PSMA4	↔	↔	↑	↔	↔	↔
CXCL9	NA	NA	NA	NA	NA	NA
LOX	NA	NA	NA	NA	NA	NA
EDNRA	↔	↓	↔	↓	↓	↓
MMP1	NA	NA	NA	NA	NA	NA
CXCL10	NA	NA	NA	NA	NA	NA
VCAN	↔	↔	↔	↑	↓	↔
ITGA2	↓	↑	↔	↑	↓	↓
CXCL13	NA	NA	NA	NA	NA	NA
CCL20	NA	NA	NA	NA	NA	NA
PLAU	↓	↓	↓	↓	↓	↓
ISG15	↑	↓	↑	↓	↑	↓
F12	↔	↔	↔	↔	↔	↔
TNFAIP6	↓	↓	↔	↔	↔	↔
PLA2G7	↔	↑	↔	↑	↔	↑
MICB	NA	NA	NA	NA	NA	NA
CXCL6	NA	NA	NA	NA	NA	NA
TNFSF4	NA	NA	NA	NA	NA	NA
KRT8	↑	↑	↑	↑	↔	↓
CXCR4	↑	↔	↔	↔	↔	↑
RIPK2	↓	↓	↔	↑	↑	↓
CCL18	NA	NA	NA	NA	NA	NA
INDO	↓	↔	↔	↔	↔	↔
BANF1	↔	↔	↑	↓	↔	↓
CXCL11	↑	↑	↓	↑	↔	↓
RSAD2	↔	↔	↓	↓	↑	↓
CCNB1	↑	↑	↑	↑	↑	↓
MIF	↓	↑	↑	↔	↓	↓
CLDN1	↑	↓	↑	↔	↔	↔
GREM1	↓	↔	↔	↑	↓	↑
MLF1IP	↑	↑	↔	↑	↔	↔
ZC3H8	↔	↔	↑	↔	↓	↔
THOC4	↔	↔	↔	↔	↔	↔
Down-regulated Genes						
LEAP2	↔	↓	↑	↓	↓	↔
CLU	↔	↓	↑	↔	↓	↓
CD59	↓	↑	↑	↔	↓	↔
LTF	↑	↓	↔	↑	↑	↓
UNC13B	↓	↓	↑	↔	↔	↓
C7	↑	↓	↑	↔	↓	↓
F13A1	↔	↔	↔	↔	↔	↔
TF	↓	↔	↔	↔	↓	↔
CXCL12	↔	↑	↑	↑	↑	↑
MPZL2	NA	NA	NA	NA	NA	NA
CD302	NA	NA	NA	NA	NA	NA
DMD	↓	↔	↔	↔	↑	↓
CDO1	↓	↑	↓	↓	↓	↑
PIGR	↓	↓	↔	↔	↔	↓
EDNRB	NA	NA	NA	NA	NA	NA
PLLP	NA	NA	NA	NA	NA	NA
CCL21	↔	↔	↔	↔	↑	↔
TFF3	↑	↓	↑	↑	↓	↑
TGFBR3	↓	↔	↓	↑	↓	↔
MAL	NA	NA	NA	NA	NA	NA
PTGER4	↓	↓	↓	↔	↔	↓
KIT	↓	↔	↓	↔	↔	↓
CFD	↑	↑	↑	↑	↑	↑
IL1R2	↔	↓	↓	↔	↓	↓
SCNN1B	↔	↑	↑	↓	↓	↓
GHR	NA	NA	NA	NA	NA	NA
MMRN1	↓	↑	↑	↓	↔	↓
MST1	↓	↓	↓	↑	↔	↔
CTSG	↔	↔	↔	↔	↔	↔
GFRA1	NA	NA	NA	NA	NA	NA
F8	↔	↑	↔	↑	↓	↓
CD8A	↔	↔	↔	↔	↔	↔
LIFR	↓	↓	↓	↓	↓	↓
PTX3	↑	↔	↓	↓	↓	↓
TLR3	↑	↑	↑	↔	↑	↑
CD36	↔	↓	↓	↔	↔	↔
P2RY14	↑	↓	↔	↔	↓	↓
IL18RAP	↑	↓	↑	↔	↔	↓
DARC	↔	↓	↔	↓	↔	↔
FOS	↑	↔	↔	↓	↔	↓
AZGP1	↔	↓	↓	↔	↓	↓
SERPINA5	NA	NA	NA	NA	NA	NA
CFH	↔	↔	↓	↑	↔	↔
GIMAP5	↓	↓	↓	↓	↓	↓
PLSCR4	↓	↑	↓	↓	↑	↑
LYVE1	↔	↔	↔	↔	↔	↔
JAM2	NA	NA	NA	NA	NA	NA
CCL28	NA	NA	NA	NA	NA	NA
CXCL17	NA	NA	NA	NA	NA	NA
AMICA1	NA	NA	NA	NA	NA	NA
VSIG2	↔	↓	↑	↓	↑	↓
SCN4B	NA	NA	NA	NA	NA	NA
