# Supplementary material for: Molecular Profiling of Multiple Human Cancers Defines an Inflammatory Cancer-Associated Molecular Pattern and Uncovers KPNA2 as a Uniform Poor Prognostic Cancer Marker
Source: PLoS One. 2013 Mar 25;8(3):e57911. doi: 10.1371/journal.pone.0057911 (PMC3607594; doi:10.1371/journal.pone.0057911)
Supplement: Table S4 — The number of Oncomine datasets showing elevation or repression of each gene in ovarian cancer. U, up. D, down. X, no change. (PDF) [file pone.0057911.s009.pdf]

Up-regulated genes		Down-regulated	
FN1	2U1D3X	LEAP2	1X
SPP1	6U	CLU	3U2D1X
STAT1	5U1D	CD59	5D1X
KPNA2	6U	LTF	1U1D3X
BST2	2U2D2X	UNC13B	3D1U1X
TNC	6U	C7	6D
COL3A1	3U1D2X	F13A1	2U2D2X
TAP1	3U3X	TF	4U2X
MYC	1U4D1X	CXCL12	1U2D3X
IL8	3U3X	MPZL2	4U1D
COL5A1	2U2D2X	CD302	6D
PSMA4	4U1D1X	DMD	1U4D
CXCL9	3U3X	CDO1	5D1X
LOX	4U1D1X	PIGR	4U2X
EDNRA	6D	EDNRB	3D3X
MMP1	3U3X	PLLP	1U3D1X
CXCL10	3U3X	CCL21	1U3D2X
VCAN	5U1X	TFF3	3U3D
ITGA2	3U2X	TGFBR3	6D
CXCL13	4U1X	MAL	6U
CCL20	3U3X	PTGER4	1U3D2X
PLAU	6U	KIT	4D2X
ISG15	4U2X	CFD	3D3X
F12	5U1X	IL1R2	3U1D2X
TNFAIP6	5U1X	SCNN1B	2U1D3X
PLA2G7	6X	GHR	6D
MICB	4U2X	MMRN1	1U3D1X
CXCL6	1U2D3X	MST1	2U2D2X
TNFSF4	1U2D3X	CTSG	2D4X
KRT8	5U1D	GFRA1	2D3X
CXCR4	5U1X	F8	3U3X
RIPK2	4U1X	CD8A	1U1D4X
CCL18	2U1D2X	LIFR	2D4X
INDO	3U3X	PTX3	6U
BANF1	2U1D2X	TLR3	4D1X
CXCL11	3U3X	CD36	2D4X
RSAD2	3U1D	P2RY14	1U3D2X
CCNB1	6U	IL18RAP	1D4X
MIF	5U1X	DARC	1U2D3X
CLDN1	1U3D	FOS	1U5D
GREM1	2U3X	AZGP1	4U2X
MLF1IP	4U	SERPINA5	2U2D2X

Up-regulated genes (continued)		Down-regulated genes (continued)	
ZC3H8	1U	CFH	4D1X
THOC4	no data	GIMAP5	1U1D3X
		PLSCR4	4D
		LYVE1	4D
		JAM2	3D1X
		CCL28	1U
		CXCL17	1U
		AMICA1	1X
		VSIG2	1X
		SCN4B	1D
